# Polygenic risk of idiopathic pulmonary fibrosis and COVID-19 severity

**DOI:** 10.1183/23120541.00978-2024

**Published:** 2025-04-14

**Authors:** Beatriz Guillen-Guio, Itahisa Marcelino-Rodriguez, José Miguel Lorenzo-Salazar, Olivia C. Leavy, Richard J. Allen, Ericka N. Pompa-Mera, SCOURGE Cohort Group, José A. Riancho, Augusto Rojas-Martinez, Pablo Lapunzina, Ángel Carracedo, Louise V. Wain, Carlos Flores

**Affiliations:** 1 Department of Population Health Sciences, University of Leicester, Leicester, UK; 2 NIHR Leicester Biomedical Research Centre, Leicester, UK; 3 Research Unit, Hospital Universitario Nuestra Señora de Candelaria, Instituto de Investigación Sanitaria de Canarias, Santa Cruz de Tenerife, Spain; 4 Preventive Medicine and Public Health Area, Universidad de La Laguna, Santa Cruz de Tenerife, Spain; 5 Genomics Division, Instituto Tecnológico y de Energías Renovables, Santa Cruz de Tenerife, Spain; 6 Unidad de Investigación Médica en Enfermedades Infecciosas y Parasitarias, Hospital de Pediatría, Centro Médico Nacional s.XXI, Instituto Mexicano del Seguro Social, Mexico City, Mexico; 7 Unidad de Investigación, Hospital de Infectología, Centro Médico Nacional “La Raza”. Instituto Mexicano del Seguro Social, Mexico City, Mexico; 8 IDIVAL, Cantabria, Spain; 9 Departamento de Medicina y Psiquiatría, Universidad de Cantabria, Cantabria, Spain; 10 Servicio de Medicina Interna, Hospital Universitario Marqués de Valdecilla, Cantabria, Spain; 11 Centro de Investigación Biomédica en Red de Enfermedades Raras, Instituto de Salud Carlos III, Madrid, Spain; 12 Tecnologico de Monterrey, The Institute for Obesity Research and Escuela de Medicina y Ciencias de la Salud, Monterrey, Mexico; 13 Instituto de Genética Médica y Molecular, Hospital Universitario La Paz-IDIPAZ, Madrid, Spain; 14 ERN-ITHACA-European Reference Network, Madrid, Spain; 15 Grupo de Medicina Xeómica, CIMUS, Universidade de Santiago de Compostela, Santiago de Compostela, Spain; 16 Instituto de Investigación Sanitaria de Santiago, Santiago de Compostela, Spain; 17 Centro Singular de Investigación en Medicina Molecular y Enfermedades Crónicas, Universidade de Santiago de Compostela, Santiago de Compostela, Spain; 18 Fundación Pública Galega de Medicina Xenómica, Centro Nacional de Genotipado, Sistema Galego de Saúde, Santiago de Compostela, Spain; 19 Centro de Investigación Biomédica en Red de Enfermedades Respiratorias, Instituto de Salud Carlos III, Madrid, Spain; 20 Facultad de Ciencias de la Salud, Universidad Fernando Pessoa Canarias, Las Palmas de Gran Canaria, Spain; 21 For a list of the SCOURGE Cohort Group members and their affiliations see the supplementary material; 22 These authors contributed equally

## Abstract

**Background:**

A shared genetic component between coronavirus disease 2019 (COVID-19) and idiopathic pulmonary fibrosis (IPF) has been described based on analyses of individual risk variants. Here we used a whole-genome polygenic risk score (PRS) approach to further evaluate age- and sex-stratified genetic overlap between IPF and severe COVID-19 to give insight into shared biological mechanisms that might both inform therapeutic strategies for both diseases.

**Methods:**

We used results from the largest genome-wide association study of clinically defined IPF risk (4125 cases/20 464 controls) and individual-level data from the SCOURGE European study of COVID-19 (5968 cases/9056 controls). We calculated IPF PRSs and assessed their association with COVID-19 severity, stratified by age and sex. We performed replication in an independent dataset of Latin-American patients (1625 cases/1887 controls). Enrichment and pathway-specific PRS analyses were performed to study biological pathways associated with COVID-19 severity.

**Results:**

IPF PRSs were significantly associated with COVID-19 hospitalisation and severe illness in Europeans and replicated in a Latin-American cohort. The strongest association was found in <60 years patients, especially among younger males (p=6.39×10^−5^). Pathway-specific PRSs analyses supported a link to cadherin and integrin signalling pathways.

**Conclusions:**

The study indicates age and sex-dependent genome-wide genetic overlap between IPF and severe COVID-19 and highlights specific shared biological mechanisms underlying both conditions. This could also imply that individuals with a high IPF genetic risk are at an overall increased risk of developing lung sequelae resulting from severe COVID-19.

## Shareable abstract

**Whole-genome polygenic risk score approach indicates age- and sex-dependent genome-wide genetic overlap between IPF and severe COVID-19, and suggests existence of shared biological mechanisms underlying pathogenesis of both diseases** https://bit.ly/3AJ8wim

## Introduction

Coronavirus disease 2019 (COVID-19) is an infectious disease caused by the acute respiratory syndrome coronavirus 2 (SARS-CoV-2). Recent studies have shown that patients hospitalised by COVID-19 can develop residual lung abnormalities, including lung fibrosis [1, 2], although biological mechanisms underlying this process remain unclear. Idiopathic pulmonary fibrosis (IPF) is a chronic, progressive, rare lung disease with a median survival of 3 years after diagnosis [3]. IPF has been shown to be an important risk factor for COVID-19 severity [4]. Additionally, studies have reported clinical and radiological similarities between severe COVID-19 and IPF [5]. Thus, there may be specific biological pathways that are common to the pathophysiology of both IPF and severe COVID-19 and could have implications for treatment of either disease.

Previous genetic studies have reported a shared causal genetic aetiology between IPF and COVID-19 severity [4, 6]. Out of the 19 common genetic variants previously reported for IPF risk, 4 of them were also associated with COVID-19 hospitalisation and severity, either increasing or decreasing disease risk, depending on the specific variant [6]. These include the most strongly associated IPF risk variant in genome-wide association studies (GWAS), located at the promoter of *MUC5B*, which displays an opposite direction of effect in IPF and COVID-19 (*i.e.* the IPF risk allele was protective for COVID-19). Interestingly, a Mendelian randomisation analysis based on 15 genome-wide significant IPF risk variants known at the time revealed that, when the *MUC5B* locus was excluded, IPF had a causal effect on COVID-19 severity [4].

Prior studies assessing the genetic overlap between IPF and COVID-19 have been based on sentinel variants associated with IPF susceptibility at the genome-wide significance level (p<5×10^−8^) in published GWAS. Here we hypothesise that the genetic overlap between both traits might be more extensive than previously reported. To date, no studies have evaluated the effect of combined whole-genome polygenic risk score (PRS) for IPF on COVID-19 severity. With the aim of identifying shared genes and biological pathways involved in the pathophysiology of both diseases, here we used a whole-genome PRS model to evaluate the genetic overlap between IPF and COVID-19 severity at the genome-wide level and to assess whether associations were age and/or sex dependent.

## Methods

### Sample and phenotype definition

To evaluate the genetic overlap between IPF and severe COVID-19, we used IPF GWAS results as the base dataset to build PRS models and applied these models to calculate PRSs for each individual in a European COVID-19 dataset (target dataset). We then tested the association of the PRSs with COVID-19 severity. We used a Latin-American COVID-19 dataset for replication purposes.

For IPF, we used publicly available summary statistic results from a large meta-GWAS of clinically defined IPF susceptibility comprising 4125 IPF cases and 20 464 population controls of European ancestry from five different studies from the United Kingdom, United States and Spain (https://github.com/genomicsITER/PFgenetics) [7]. All IPF cases were diagnosed according to the American Thoracic Society and European Respiratory Society guidelines [8, 9].

For COVID-19, we used individual-level genetic data (single-nucleotide polymorphism (SNP) arrays) from 11 939 individuals from the Spanish Coalition to Unlock Research on Host Genetics on COVID-19 (SCOURGE) study [10]. Individuals were diagnosed as COVID-19-positive either through a PCR-based test (81.7% of cases) or based on local clinical evaluations (3.4%) and laboratory methods (14.2% *via* antibody tests; 0.7% *via* other microbiological tests). All patients were recruited from 34 Spanish centres in 25 cities between March and December 2020 and had not been vaccinated at the time of sample collection [10]. SCOURGE samples and data were collected by the participating centres through their respective biobanks and clinical registries. Demographic, epidemiological, and clinical data was collected and managed using research electronic data capture (REDCap) tools hosted at Centro de Investigación Biomédica en Red (CIBER) [11, 12] (supplementary notes). We considered three previously defined COVID-19 phenotypes as cases to evaluate the response to COVID-19 infection: hospitalisation, severe illness and critical illness. Hospitalised patients encompassed all individuals admitted to the hospital with a confirmed COVID-19 diagnosis. The “severe illness” category comprised patients falling in scales 3 and 4 of the severity scale defined by the SCOURGE consortium (table 1) [10], whereas “critical illness” included patients in scale 4. We assigned as controls all other patients with COVID-19 not satisfying the case criteria for each of the three categories, plus a total of 5943 population controls from the Spanish DNA biobank (www.bancoadn.org) and GR@ACE consortium (Genome Research at Fundació ACE) [13].

**TABLE 1:** Classification of patients with COVID-19 from SCOURGE into levels of severity

	Clinical findings	n
Severity 0 (asymptomatic)	Asymptomatic	582
Severity 1 (mild)	With symptoms, but without pulmonary infiltrates or need of oxygen therapy	2689
Severity 2 (moderate)	With pulmonary infiltrates affecting <50% of the lungs or need of supplemental oxygen therapy	2099
Severity 3 (severe)	Hospitalised with any of the following criteria: *P*_aO_2__<65 mmHg or *S*_aO_2__<90% *P*_aO_2__/*F*_IO_2__<300 *S*_aO_2__/*F*_IO_2__<440 Dyspnoea Respiratory frequency ≥22 rpm Infiltrates affecting >50% of the lungs	2379
Severity 4 (critical)	Admission to the ICU or need of mechanical ventilation (invasive or noninvasive)	1128

COVID-19: coronavirus disease 2019; SCOURGE: Spanish Coalition to Unlock Research on Host Genetics on COVID-19; *P*_aO_2__: partial pressure of oxygen in arterial blood; *F*_IO_2__: fraction of inspired oxygen; *S*_aO_2__: saturation of oxygen in arterial blood; rpm: respirations per min; ICU: intensive care unit. Data were extracted from Cruz e*t al.* [10].

For the replication stage, we used individual-level genetic data from an independent dataset of Latin-American individuals from the SCOURGE study recruited from March 2020 to July 2021 [14]. The sample comprised a total of 1625 hospitalised patients with COVID-19 as cases and 1887 nonhospitalised COVID-19 patients as controls. Data from Latin-American SCOURGE individuals were also collected and managed using the REDCap electronic data capture tool.

The SCOURGE study was performed in accordance with the Declaration of Helsinki. Study samples and data were collected by the participating centres, through their respective biobanks after informed consent, with the approval of the respective Ethic and Scientific Committees. The SCOURGE study was approved by the Galician Ethical Committee (ref. 2020/197). The recruitment of patients from Mexico City was approved by the National Ethical and Research Committee from the Mexican Institute of Social Security (protocol R-2020-785-082). The recruitment of patients from Monterrey was approved by the Ethics and Research Committee of the School of Medicine of the Tecnológico de Monterrey (protocol P000396-GenoCOVID-CEIC-CR002). Additionally, the Federal Commission for the Protection against Sanitary Risks approved authorisation for the international collaboration (authorisation 203301410A0265/2020). For IPF, we used only publicly available human data. Detailed information about both IPF and COVID-19 datasets can be found in the original publications describing the studies [7, 10].

### Genotyping and variant imputation

Samples were genotyped using the Axiom Spain Biobank Array (Thermo Fisher Scientific). Genotyping and quality control procedures have been previously described [10]. Principal components (PCs) were calculated conducted using a linkage disequilibrium (LD)-pruned subset of independent genotyped SNPs (r^2^ < 0.1) to reduce the effects of population stratification in the analysis. SNP imputation was performed using the TOPMed version r^2^ reference panel (GRCh38) in the TOPMed Imputation Server [15, 16] and variants with a minor allele frequency (MAF) <1% or low imputation quality (R^2^<0.3) were filtered out. Genotyping, quality control and imputation procedures for the replication cohort were conducted in a manner mirroring that of the SCOURGE European study [14]. European and Latin-American genetic ancestry were inferred using ADMIXTURE [17] with populations from the 1000 Genomes Project serving as reference data [10, 14].

### PRS modelling and statistical analyses

We used PRSice-2 [18] to calculate PRSs for each individual in the COVID-19 dataset. PRSs were estimated as the sum of risk alleles from the IPF GWAS variants weighted by their effect sizes:

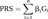


Where β is the weight or log odds ratio (OR) for variant i, G is the number of risk alleles carried at variant i and n is the number of variants included in the score. Briefly, PRSice-2 performs clumping to remove SNPs in high LD (parameters were set at threshold R^2^= 0.1 for a 250-kb window for clumping), derives PRSs at different p-value thresholds in the base GWAS, tests the association of each of the PRS in the target dataset and predicts the best model fit of the phenotype. The selection of the optimal model relies on the association significance of the model. Therefore, for the various severity phenotypes examined (hospitalisation, severe illness and critical illness, both with and without stratification by sex and age), the number of SNPs included in the optimal model may differ. This approach was also applied to the replication stage.

All PRSs were standardised as z-scores using the formula:
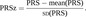


Binomial logistic regression was performed with R v.4.0.3 (R) [19] to assess the association of the individual PRS with COVID-19 hospitalisation, severe illness and critical illness separately. To account for the ascertainment bias resulting from restricting analyses to a specific population (*i.e.* hospitalised patients tested for COVID-19), we considered a prevalence of COVID-19 hospitalisation of 0.5% in the PRS analyses, in line with previous estimates in the SCOURGE cohort [10]. Prevalence of severe illness (59% of hospitalised patients in the SCOURGE cohort) and critical illness (19% of hospitalisations) were estimated to be 0.295% and 0.095%, respectively [10]. We also performed stratifications by sex (male and female), age (<60 years and ≥60 years; to align with previous studies [4, 10, 20] and sex and age (male <60 years, male ≥60 years, female <60 years and female ≥60 years). All models were adjusted for the first 10 PCs for genetic ancestry and, whenever necessary, for age and sex. Significance in the discovery stage was declared at p≤1.8×10^−3^ to control type-I error in the 27 comparisons (Bonferroni correction for nine models for three outcomes). For the replication stage, only the COVID-19 hospitalisation categories that were significant in the discovery stage were tested and an appropriate multiple testing correction was applied.

### Sensitivity analyses

To confirm the robustness of the PRS estimates in the significant comparisons, we calculated the individual PRSs using an alternative method with megaPRS [21]. Briefly, MegaPRS used the IPF GWAS results to calculate different prediction models using variational Bayes and identified the best prediction model. This model was used to calculate PRSs in the severe COVID-19 cohort, followed by association analyses with the different COVID-19 severity phenotypes. We also performed analyses excluding patients with a clinical history of chronic respiratory diseases (including COPD, asthma, pulmonary fibrosis, pulmonary hypertension and lung cancer) or using only at-risk controls (*i.e.* nonhospitalised patients with COVID-19) as the control group. Additionally, we studied the predictive capacity of the PRSs on 90-day COVID-19 mortality (Bonferroni-corrected p-value threshold of 0.05/9=5.6×10^−3^). Finally, we compared the effect of whole-genome PRS calculated from genome-wide variants with a PRS obtained from only the 19 previously published genome-wide significant IPF common risk variants (sentinels PRS) (supplementary table S1) [7]. These comparisons were also tested excluding the *MUC5B* locus, the strongest common genetic risk factor for IPF both in terms of significance of association and effect size, to evaluate its impact on the PRS models. This was done by either excluding the *MUC5B* promoter variant (supplementary table S1) from the sentinels PRS model or excluding all the variants in the ±1-Mb flanking regions from the whole-genome PRS model to prevent the inclusion of proxy LD variants of the sentinel SNP.

### Pathway analyses

With the aim of identifying potential biological mechanisms linked to both IPF and COVID-19 severity, we investigated whether the variants included in the IPF PRS models that significantly associated with COVID-19 severity were involved in particular signalling mechanisms. We first used the online data tool GREAT [22] to obtain the list of genes that were experimentally or computationally linked through regulatory annotations to the genetic variants included in the best PRS model of COVID-19 hospitalisation for the entire study sample (*i.e.* the variants selected by PRSice-2 for providing the best p-value threshold; table 2). We then performed a gene set enrichment analysis with ShinyGO v.0.77 [23] to assess whether the obtained gene list was enriched in genes involved in certain biological pathways (false discovery rate (FDR) < 0.05). To identify which specific pathways were related to COVID-19 hospitalisation, we used PRSice-2 to calculate pathway-specific PRSs for the top five pathways over-represented in the gene list. We included only the variants associated with each of the five pathways separately in the PRS computation and then studied the PRSs association with COVID-19 hospitalisation. Significance was declared at p<0.05.

**TABLE 2:** Association of idiopathic pulmonary fibrosis (IPF) polygenic risk score (PRS) models with COVID-19 severity

Category	Covariates	Cases/controls^#^	PT	Num_SNP	OR (95% CI)	p-value^¶^
**Hospitalisation**						
All individuals	Age, sex, 10 PC	5968/9056	2.10×10^−3^	2939	1.08 (1.04–1.12)	**7.90×10^−5^**
Males	Age, 10 PC	3441/3958	7.85×10^−3^	8686	1.09 (1.04–1.16)	**1.28×10^−3^**
Females	Age, 10 PC	2525/5096	2.25×10^−3^	3083	1.08 (1.02–1.14)	6.44×10^−3^
≥60 years	Sex, 10 PC	4328/2355	7.50×10^−4^	1330	1.06 (1.01–1.12)	0.026
<60 years	Sex, 10 PC	1607/6457	4.70×10^−3^	5660	1.12 (1.06–1.19)	**3.44×10^−5^**
≥60 years, males	10 PC	2436/878	7.85×10^−3^	8686	1.06 (0.98–1.15)	0.123
≥60 years, females	10 PC	1892/1477	7.50×10^−4^	1330	1.09 (1.01–1.17)	0.021
<60 years, males	10 PC	987/2904	4.70×10^−3^	5660	1.16 (1.08–1.25)	**6.39×10^−5^**
<60 years, females	10 PC	619/3551	0.070	51 746	1.13 (1.04–1.23)	6.04×10^−3^
**Severe illness**						
All individuals	Age, sex, 10 PC	3502/10 846	6.65×10^−3^	7566	1.08 (1.04–1.13)	**2.57×10^−4^**
Males	Age, 10 PC	2109/4897	6.40×10^−3^	7331	1.08 (1.02–1.14)	6.05×10^−3^
Females	Age, 10 PC	1393/5949	0.014	13 949	1.09 (1.02–1.16)	7.93×10^−3^
≥60 years	Sex, 10 PC	2543/3834	0.014	14 289	1.05 (1.00–1.11)	0.057
<60 years	Sex, 10 PC	959/7012	3.30×10^−3^	4198	1.14 (1.07–1.22)	**1.53×10^−4^**
≥60 years, males	10 PC	1526/1631	1.00	308 260	1.06 (0.99–1.14)	0.095
≥60 years, females	10 PC	1017/2203	0.014	13 949	1.08 (1.00–1.17)	0.047
<60 years, males	10 PC	583/3266	5.05×10^−3^	6009	1.16 (1.06–1.27)	**9.98×10^−4^**
<60 years, females	10 PC	376/3746	0.073	53 482	1.15 (1.03–1.28)	0.010
**Critical illness**						
All individuals	Age, sex, 10 PC	1124/13 224	0.014	13 828	1.12 (1.06–1.20)	**2.49×10^−4^**
Males	Age, 10 PC	815/6191	1.90×10^−3^	2728	1.12 (1.04–1.21)	2.10×10^−3^
Females	Age, 10 PC	309/7033	0.053	41 838	1.23 (1.10–1.39)	**3.67×10^−4^**
≥60 years	Sex, 10 PC	776/5601	0.014	13 949	1.13 (1.04–1.21)	2.36×10^−3^
<60 years	Sex, 10 PC	348/7623	0.047	37 678	1.16 (1.04–1.30)	6.83×10^−3^
≥60 years, males	10 PC	554/2603	3.10×10^−3^	3961	1.15 (1.05–1.26)	2.92×10^−3^
≥60 years, females	10 PC	222/2998	0.053	41 863	1.18 (1.03–1.36)	0.017
<60 years, males	10 PC	261/3588	7.45×10^−3^	8328	1.11 (0.98–1.26)	0.090
<60 years, females	10 PC	87/4035	0.129	84 188	1.44 (1.16–1.78)	**8.97×10^−4^**

COVID-19: coronavirus disease 2019; PC: principal component; PT: best-fit p-value threshold; Num_SNP: number of single-nucleotide polymorphisms included in the best model. ^#^: A total of 281 individuals had missing data for sex (n=4) and age (n=277). **^¶^**: Association results for the best prediction model resulting from PRSice-2 in each category (the PRS includes all variants reaching the best p-value threshold in the IPF genome-wide association studies). p-values showing a significant association after Bonferroni correction (p≤1.85×10^−3^) are highlighted in bold.

## Results

The COVID-19 SCOURGE dataset comprised a total of 15 024 individuals of European genetic ancestry. Sample sizes for each COVID-19 phenotype and stratification category are summarised in table 2. A total of 308 260 clumped variants from the IPF meta-GWAS were used for PRS analyses with PRSice-2.

IPF whole-genome PRS were found to be associated with COVID-19 hospitalisation (OR 1.08, 95% confidence interval (CI) 1.04–1.12; p=7.90×10^−5^), severe illness (OR 1.08, 95% CI 1.04–1.13; p=2.57×10^−4^) and critical illness (OR 1.12, 95% CI 1.06–1.20; p=2.49×10^−4^). When the COVID-19 hospitalisation PRS was divided into quartiles, individuals in the highest PRS quartile had a 24% increased risk (OR 1.24, 95% CI 1.11–1.39) of hospitalisation when compared with the reference group in the lowest quartile (supplementary figure S1). For severe illness and critical illness, the highest PRS quartile was associated with an increased risk of 19% (OR 1.19, 95% CI 1.05–1.34) and 34% (OR 1.34, 95% CI 1.12–1.59), respectively. Stratifying by age and/or sex, the strongest association was found in hospitalised younger individuals (<60 years), especially among younger males (table 2 and supplementary figure S2). Among these categories, the lowest number of IPF genetic variants that were needed to calculate the best performing whole-genome PRS was 2939 variants, all of them reaching a p-value threshold of 2.10×10^−3^ in the GWAS of IPF (table 2 and supplementary figure S3). IPF PRSs association results in the four significant hospitalisation categories were replicated in an independent cohort of Latin-American patients with COVID-19. Following Bonferroni correction (p=0.05/4=0.0125), replication was observed for males, <60 years individuals and <60 years males, while results were nominally significant when all individuals were considered (table 3).

**TABLE 3:** Replication of the association of idiopathic pulmonary fibrosis polygenic risk score (PRS) with COVID-19 hospitalisation in Latin-American people

COVID-19 hospitalisation category^#^	PT	OR (95% CI)	p-value^¶^
All	0.432	1.17 (1.03–1.32)	0.016
Males	0.035	1.25 (1.07–1.45)	**3.82×10^−3^**
<60 years	0.064	1.18 (1.05–1.33)	**5.65×10^−3^**
<60 years, males	0.035	1.23 (1.05–1.44)	**8.53×10^−3^**

COVID-19: coronavirus disease 2019; PT: best-fit p-value threshold. ^#^: Results for the categories where the best-fit PRSice PRS showed a significant association with COVID-19 hospitalisation after multiple testing correction in the discovery stage. Significance was declared at p=0.0125 after multiple testing correction. ^¶^: Association results for the best prediction model resulting from PRSice-2 in each category.

PRSs association results using megaPRS estimations supported the robustness of associations of the IPF whole-genome PRS with COVID-19 hospitalisation and severe illness (supplementary table S2). MegaPRS association results for critical illness were inconsistent, potentially attributed to the limited sample size available for this particular phenotype. This precludes us from drawing any conclusions related to critical illness categories. *Post hoc* analyses excluding 892 patients with a clinical history of chronic respiratory diseases supported the robustness of the results in the significant hospitalisation categories (supplementary table S3). When comparing hospitalised COVID-19 patients with individuals with COVID-19 who were not hospitalised, the effect sizes and directions of the association results were consistent across all categories (supplementary table S4). However, statistical significance after Bonferroni correction (p=0.0125) was only observed for <60-year-old individuals, possibly due to the reduced sample size after removing population controls. IPF whole-genome PRSs did not show an association with 90-day COVID-19 mortality in the SCOURGE study for any of the best models in each category (supplementary table S5).

The exclusion of the *MUC5B* locus (promoter variant and variants in the ±1-Mb flanking regions) from the IPF PRS modelling resulted in a stronger association of the IPF whole-genome PRS with COVID-19 hospitalisation (supplementary table S6). When using sentinels PRSs calculated from the previously known IPF risk variants, the association with COVID-19 hospitalisation was only significant for models where the *MUC5B* locus had been excluded (supplementary table S6). We next compared the results of the IPF whole-genome PRS to those of the sentinels PRS excluding the *MUC5B* locus. When considering all individuals or all younger (<60 years old) individuals, the effect for COVID-19 hospitalisation was larger when whole-genome PRSs were used (supplementary table S6). However, among males, the effect size was bigger when excluding *MUC5B* from the sentinels PRS. The effect did not differ in <60-year-old males.

A gene set enrichment analysis was performed to identify the biological processes that may be related to both IPF and COVID-19 hospitalisation. We focused on the 3471 genes linked through experimental and computational regulatory annotations by GREAT to the 2939 SNPs included in the whole-genome PRS model associated with COVID-19 hospitalisation in the whole sample (table 2). Results showed a significant enrichment in genes involved in cadherin, Wnt and integrin signalling pathways, among others (supplementary figure S4a). Results were similar when the 19 IPF common loci (sentinels ±1 Mb) were excluded from the analysis (supplementary figure S4b) and when we used the list of 5660 SNPs used to calculate the best-fit PRS associated with COVID-19 hospitalisation in <60-year-old males (table 2 and supplementary figure S5). Pathway-specific PRSs analyses revealed that the PRS including the integrin pathway variants was associated at the nominal level with COVID-19 hospitalisation in patients younger than 60 years (p=7.0×10^−3^) (supplementary table S7). Additionally, the cadherin pathway PRS was nominally associated with COVID-19 hospitalisation in males (p=0.028) (supplementary table S7). No significant associations were observed for the remaining pathways.

## Discussion

We performed a PRS-based association analysis to study the genetic overlap between IPF and the severity of COVID-19. Our results show that IPF PRSs obtained from genome-wide variants were significantly associated with COVID-19 hospitalisation in patients of European ancestry, suggesting that IPF and severe COVID-19 could share certain biological mechanisms. These findings were also replicated in patients with COVID-19 from Latin America. Our results were also robust when using an alternate method to estimate the PRSs, excluding patients with previously reported chronic respiratory diseases and using nonhospitalised patients with COVID-19 as controls (instead of relying on population-based controls with uncertain SARS-CoV-2 exposure at the time of data collection). IPF PRSs were not associated with COVID-19 90-day mortality, indicating that COVID-19 severity and mortality may be governed by distinct genetic factors, consistent with previous findings [24].

The strongest associations of IPF PRS models with COVID-19 were found for the hospitalised patients who were younger (<60 years old), particularly in younger males. These results agree with the findings of Nakanishi *et al.* [25], who reported that the effects of carrying common genetic risk factors of severe COVID-19 were stronger in individuals younger than 60 years. Cruz *et al.* [10] also showed that the genetic risk score combining the main COVID-19 genetic risk factors had a higher predictive capability among the <60-year-old males from the SCOURGE study. This support that the presence of genetic risk factors that affect disease severity would be more evident among younger patients, while severe COVID-19 in older individuals (≥60 years old) might be more influenced by nongenetic factors such as comorbidities and immunological defects [26].

The existence of genetic factors shared between IPF and COVID-19 had already been described by several studies [4, 6]. However, here we suggest that the number of overlapping variants underlying IPF risk and severe COVID-19 could be larger than previously reported. In fact, the sentinels PRS calculated based on only the previously reported IPF risk variants was not significantly associated with severe COVID-19 in our data. However, when the strongest known IPF genetic risk factor at *MUC5B* was excluded from the sentinels PRS model, the effect on COVID-19 hospitalisation was stronger, as previously reported by Fadista *et al.* [4]. This could be explained by the different direction of effect that the *MUC5B* variant has for IPF and COVID-19 [4, 6]. After excluding the *MUC5B* locus, the effect for COVID-19 hospitalisation was larger in all and <60-year-old patients when using whole-genome PRSs compared with sentinels PRS results. Nevertheless, the effect on COVID-19 hospitalisation was similar with both approaches in males and younger males. This could also be attributed to the arbitrary LD clumping method that was used to select variants to be included in the whole-genome PRS models, compared with the refined selection of the 19 IPF risk variants involved in the sentinels PRS models.

Given that an increased genetic risk for IPF is associated with a higher risk of severe COVID-19, and that severe COVID-19 also increases risk of post-infection lung sequelae among survivors [1], individuals at high IPF genetic risk could be at an overall increased risk of developing post-COVID-19 lung fibrotic sequelae. This is relevant not only for patients already diagnosed with IPF but also for individuals at higher genetic risk of IPF who have not developed the disease. Our findings suggest at least a 10% increase in the risk of developing severe COVID-19 risk per sd increase in the IPF PRS. With the growing interest in utilising PRS in clinical applications, even modest increases in risk could lead to significant implications in public health. This underscores the need to understand wider implications of PRSs across different phenotypes. Moreover, uncovering genetic overlaps might highlight potential shared targets for drug repurposing.

Our study reinforces the existence of biological mechanisms shared between IPF and severe COVID-19 pathogenesis. The gene set enrichment and pathway-specific PRS analyses, focusing on the variants used to calculate the IPF PRS models significantly associated with COVID-19 severity, suggest that integrin and cadherin signalling pathways, previously related to fibrosis and lung repair processes, could have a role in COVID-19 severity. These could be of special interest among young (<60 years) patients and males based on our results. Integrins are considered key regulators during fibrogenesis, and integrin inhibitors are currently being investigated as antifibrotic strategies for IPF treatment, including an inhaled inhibitor of αvβ6 integrin [27, 28]. Additionally, several studies have reported the role of integrins in facilitating the cellular entry of SARS-CoV-2 [29, 30]. Therefore, this pathway could have significant potential as a key target for the treatment of both SARS-CoV-2 infection and post-COVID-19 pulmonary fibrosis. Signalling through cadherins has also been suggested to be involved in lung fibrosis [31]. Cadherin-11 may play an important role in the pathogenesis of IPF, possibly through the regulation of epithelial to mesenchymal transition (EMT) in alveolar epithelial cells [32], again pointing to this protein activity as a potential therapeutic target. Recent studies suggest that the EMT process could also be key in post-COVID-19 lung fibrosis [33].

A limitation of our study is the substantial amount of missing data in a significant proportion of individuals, which prevented us from incorporating adjustments for other COVID-19 severity risk factors, such as smoking status, without a marked reduction in our sample size. Future studies using independent datasets should consider and address this limitation in their model design. Larger samples sizes will also be needed to further evaluate the intriguing association between the IPF PRS and critical COVID-19 in females, particularly those under 60 years, where it showed a larger effect size. Further investigations that incorporate association results from variants on the sex chromosomes are also warranted. Functional experiments, including cell-specific assays, will be essential to elucidate the specific roles of the cadherin and integrin signalling pathways in the pathophysiology of both IPF and severe COVID-19. Among the strengths, it is worth highlighting that COVID-19 participants of SCOURGE were recruited before vaccines were widely available for the community, reducing potential biases caused by disease severity misclassification. Furthermore, contrary to previous studies, the whole-genome PRS approach enables inclusion of genetic variants associated with IPF that have not yet been reported due to limitations of statistical power and strict significance thresholds necessitated by multiple testing corrections. Moreover, the replication of our findings in a Latin-American cohort suggests that the score approach is likely generalisable across ancestries.

In summary, the use of a whole-genome PRS approach supported the existence of thousands of shared common genetic risk factors underlying the pathogenesis of both IPF and severe COVID-19. This comprehensive approach was more effective in capturing the severity of COVID-19 compared with an IPF-sentinels-only approach. Furthermore, we observed that the association of the polygenic risks of IPF with COVID-19 hospitalisation were age- and sex-dependent. Taken together, results suggest that individuals at higher risk of IPF could be at an increased risk of developing post-COVID-19 lung fibrosis. Identifying individuals at higher genetic risk may benefit from earlier and more frequent screenings, allowing for early detection and timely intervention. Additionally, our results suggest that overlapping variants could be involved in biological processes such as integrin and cadherin signalling pathways. This provides an improved comprehension of the mechanisms underlying COVID-19 severity, which is crucial to provide valuable insights for shaping future therapeutic strategies in managing COVID-19. Further studies will be needed to evaluate this possibility.

## Supplementary material

10.1183/23120541.00978-2024.Supp1 **Please note:** supplementary material is not edited by the Editorial Office, and is uploaded as it has been supplied by the author.
Supplementary material 00978-2024.SUPPLEMENT

## Data Availability

The datasets generated and/or analysed during the current study are available from the corresponding author on reasonable request. IPF genome-wide summary statistics are openly available and can be obtained from https://github.com/genomicsITER/PFgenetics.
