# Supplementary material for: Polygenic risk of idiopathic pulmonary fibrosis and COVID-19 severity
Source: ERJ Open Res. 2025 Apr 14;11(2):00978-2024. doi: 10.1183/23120541.00978-2024 (PMC12004260; doi:10.1183/23120541.00978-2024)
Supplement: Supplementary file 1 [file 00978-2024.SUPPLEMENT.pdf]

Polygenic risk of idiopathic pulmonary fibrosis and COVID-19 severity

Supplementary material

Beatriz Guillen-Guio*, Itahisa Marcelino-Rodriguez, Jose Miguel Lorenzo-Salazar, Olivia C Leavy, Richard J Allen, Ericka N. Pompa-Mera, Scourge Cohort Group, José A. Riancho, Augusto Rojas-Martinez, Pablo Lapunzina, Ángel Carracedo, Louise V Wain#, Carlos Flores#

*Corresponding Author. *Email:* bgg4@leicester.ac.uk
#Equal contribution as senior authors

Contents

Supplemental Note....2
Supplementary Tables....2
Supplementary Figures....6
References....11
Banner Scourge....12
  SCOURGE Cohort Group Members....12
  SCOURGE Cohort Group Affiliations....13

Supplemental Note

Research electronic data capture (REDCap) is a secure, web-based software platform designed to support data capture for research studies, providing 1) an intuitive interface for validated data capture; 2) audit trails for tracking data manipulation and export procedures; 3) automated export procedures for seamless data downloads to common statistical packages; and 4) procedures for data integration and interoperability with external sources [1, 2].

Supplementary Tables

Table S1. IPF sentinel variants

Locus	SNP ID	Chr.	Position (hg38)	Effect allele	Non-effect allele	OR [95% CI]	P-value[f]
KIF15	rs141979279[a]	3	44816639	C	T	1.50 [1.33,1.70]	$1.21x10^{-10}$
TERC	rs10936601[b]	3	169810661	C	T	0.79 [0.74,0.84]	$2.10x10^{-15}$
FAM13A	rs2013701	4	88963935	G	T	1.25 [1.18,1.32]	$4.60x10^{-16}$
TERT	rs7725218	5	1282299	G	A	1.41 [1.33,1.50]	$4.90x10^{-32}$
DSP	rs2076295	6	7562999	G	T	1.49 [1.41,1.57]	$1.50x10^{-48}$
MAD1L1	rs12699415	7	1869843	A	G	1.27 [1.20,1.34]	$7.85x10^{-18}$
ZKSCAN1	rs2897075	7	100032719	T	C	1.30 [1.23,1.37]	$1.77x10^{-21}$
DEPTOR	rs28513081	8	119921886	A	G	1.20 [1.13,1.27]	$1.22x10^{-9}$
10q25.1	rs79684490	10	109470103	A	G	1.40 [1.24,1.57]	$3.52x10^{-8}$
MUC5B	rs35705950	11	1219991	T	G	5.06 [4.69,5.47]	$9.09x10^{-418}$
ATP11A	rs12585036[c]	13	112881427	C	T	1.29 [1.21,1.38]	$5.99x10^{-14}$
IVD	rs59424629	15	40428343	T	G	1.27 [1.21,1.34]	$4.98x10^{-19}$
KNL1	rs12912339	15	40639510	A	G	1.30 [1.21,1.39]	$7.41x10^{-13}$
AKAP13	rs62023891	15	85553985	A	G	1.18 [1.12,1.25]	$1.32x10^{-8}$
NPRL3	rs74614704	16	112241	A	G	1.49 [1.34,1.67]	$2.57x10^{-12}$
17q21.31	rs3785884[d]	17	45980229	G	A	1.40 [1.30,1.51]	$2.53x10^{-20}$
DPP9	rs35574495[e]	19	4686976	G	T	0.80 [0.75,0.86]	$1.08x10^{-9}$
STMN3	rs112087793	20	63652817	C	T	1.34 [1.21,1.48]	$1.09x10^{-8}$
RTEL1	rs41308092	20	63693038	A	G	1.75 [1.45,2.10]	$3.13x10^{-9}$

[a]Proxy for rs78238620 (r^2=1); [b]Proxy for rs12696304 (r^2=0.99); [c]Proxy for rs9577395 (r^2=0.98); [d]Proxy for rs2077551 (r^2=0.83); [e]Proxy for rs12610495 (r^2=0.36); [f]Significance of the association in the GWAS of IPF susceptibility [3]. Linkage disequilibrium (r^2) proxies were defined using 1000 genomes European sample data. Data for the *SPDL1* locus in SCOURGE was no available given the rarity (<1%) of the IPF risk variant in the general population [4].

Table S2. IPF PRS associations with COVID-19 severity using alternative PRS estimation methods

COVID-19 severity	Category	PRSice-2				megaPRS	
		PT	Num_SNP	OR (95%CI)	P-Value*	OR (95%CI)	P-Value
Hospitalisation	All individuals	$2.10x10^{-3}$	2,939	1.08 (1.04,1.12)	**$7.90x10^{-5}$**	1.07 (1.03,1.11)	**$1.27x10^{-3}$**
	Males	$7.85x10^{-3}$	8,686	1.09 (1.04,1.16)	**$1.28x10^{-3}$**	1.08 (1.02,1.14)	$9.10x10^{-3}$
	Females	$2.25x10^{-3}$	3,083	1.08 (1.02,1.14)	$6.44x10^{-3}$	1.05 (0.99,1.11)	0.088
	≥60 years	$7.50x10^{-4}$	1,330	1.06 (1.01,1.12)	0.026	1.05 (0.99,1.10)	0.105
	<60 years	$4.70x10^{-3}$	5,660	1.12 (1.06,1.19)	**$3.44x10^{-5}$**	1.10 (1.04,1.16)	**$1.17x10^{-3}$**
	≥60 years Males	$7.85x10^{-3}$	8,686	1.06 (0.98,1.15)	0.123	1.04 (0.96,1.13)	0.310
	≥60 years Females	$7.50x10^{-4}$	1,330	1.09 (1.01,1.17)	0.021	1.05 (0.97,1.12)	0.217
	<60 years Males	$4.70x10^{-3}$	5,660	1.16 (1.08,1.25)	**$6.39x10^{-5}$**	1.13 (1.05,1.21)	**$1.55x10^{-3}$**
	<60 years Females	0.070	51,746	1.13 (1.04,1.23)	$6.04x10^{-3}$	1.06 (0.97,1.15)	0.209
Severe illness	All individuals	$6.65x10^{-3}$	7,566	1.08 (1.04,1.13)	**$2.57x10^{-4}$**	1.06 (1.02,1.11)	$4.80x10^{-3}$
	Males	$6.40x10^{-3}$	7,331	1.08 (1.02,1.14)	$6.05x10^{-3}$	1.09 (1.03,1.15)	$4.20x10^{-3}$
	Females	0.014	13,949	1.09 (1.02,1.16)	$7.93x10^{-3}$	1.03 (0.96,1.09)	0.424
	≥60 years	0.014	14,289	1.05 (1.00,1.11)	0.057	1.02 (0.97,1.07)	0.487
	<60 years	$3.30x10^{-3}$	4,198	1.14 (1.07,1.22)	**$1.53x10^{-4}$**	1.14 (1.06,1.22)	**$1.79x10^{-4}$**
	≥60 years Males	1.00	308,260	1.06 (0.99,1.14)	0.095	1.03 (0.96,1.10)	0.445
	≥60 years Females	0.014	13,949	1.08 (1.00,1.17)	0.047	1.00 (0.93,1.08)	0.906
	<60 years Males	$5.05x10^{-3}$	6,009	1.16 (1.06,1.27)	**$9.98x10^{-4}$**	1.18 (1.08,1.29)	**$2.02x10^{-4}$**
	<60 years Females	0.073	53,482	1.15 (1.03,1.28)	0.010	1.08 (0.97,1.20)	0.164
Critical illness	All individuals	0.014	13,828	1.12 (1.06,1.20)	**$2.49x10^{-4}$**	1.08 (1.01,1.15)	0.017
	Males	$1.90x10^{-3}$	2,728	1.12 (1.04,1.21)	$2.10x10^{-3}$	1.05 (0.98,1.13)	0.188
	Females	0.053	41,838	1.23 (1.10,1.39)	**$3.67x10^{-4}$**	1.14 (1.01,1.27)	0.029
	≥60 years	0.014	13,949	1.13 (1.04,1.21)	$2.36x10^{-3}$	1.10 (1.02,1.19)	0.016
	<60 years	0.047	37,678	1.16 (1.04,1.30)	$6.83x10^{-3}$	1.04 (0.93,1.16)	0.525
	≥60 years Males	$3.10x10^{-3}$	3,961	1.15 (1.05,1.26)	$2.92x10^{-3}$	1.07 (0.97,1.17)	0.173
	≥60 years Females	0.053	41,863	1.18 (1.03,1.36)	0.017	1.17 (1.02,1.34)	0.025
	<60 years Males	$7.45x10^{-3}$	8,328	1.11 (0.98,1.26)	0.090	1.01 (0.89,1.15)	0.837
	<60 years Females	0.129	84,188	1.44 (1.16,1.78)	**$8.97x10^{-4}$**	1.09 (0.88,1.34)	0.426

*Association results for the best prediction model resulting from PRSice-2. P-values showing a significant association after Bonferroni correction ($p \leq 1.85x10^{-3}$) are highlighted in bold. PC: Principal Components, PT: Best fit P-value Threshold; Num_SNP: Number of SNPs included in the best model.

Table S3. Association of IPF whole-genome PRS with COVID-19 hospitalisation in the entire sample *vs.* after excluding patients with clinical history of chronic respiratory diseases

COVID-19 hospitalisation category#	All individuals (Nt=15,024)			Excluding chronic diseases (Nt=14,132) †		
	PT	OR (95% CI)	P-value*	PT	OR (95% CI)	P-value*
All	$2.10x10^{-3}$	1.08 (1.04,1.12)	**$7.90x10^{-5}$**	$2.30x10^{-3}$	1.09 (1.04,1.13)	**$4.31x10^{-5}$**
Males	$7.85x10^{-3}$	1.09 (1.04,1.16)	**$1.28x10^{-3}$**	$7.85x10^{-3}$	1.10 (1.04,1.17)	**$7.89x10^{-4}$**
<60 years	$4.70x10^{-3}$	1.12 (1.06,1.19)	**$3.44x10^{-5}$**	$4.70x10^{-3}$	1.13 (1.07,1.20)	**$2.62x10^{-5}$**
<60 years Males	$4.70x10^{-3}$	1.16 (1.08,1.25)	**$6.39x10^{-5}$**	$4.70x10^{-3}$	1.17 (1.08,1.26)	**$7.08x10^{-5}$**

#Results for the categories where the best-fit PRSice PRS showed a significant association with COVID-19 hospitalisation after multiple tests correction in the discovery stage. P-values showing a significant association after Bonferroni correction (4 tests, p≤0.013) are highlighted in bold. *Association results for the best prediction model resulting from PRSice-2 in each category. †A total of 892 patients with chronic respiratory diseases were excluded. Nt: total sample size; PT: Best fit P-value Threshold.

Table S4. Association of IPF whole-genome PRS with COVID-19 hospitalisation including or excluding population controls

COVID-19 hospitalisation category#	Non-hospitalised COVID-19 + population controls				Non-hospitalised COVID-19 controls			
	PT	Cases/Controls*	OR (95% CI)	P-value	PT	Cases/Controls*	OR (95% CI)	P-value
All	$2.10x10^{-3}$	5,968/9,056	1.08 (1.04,1.12)	**$7.90x10^{-5}$**	$2.30x10^{-3}$	5,968/3,382	1.06 (1.01,1.12)	0.020
Males	$7.85x10^{-3}$	3,441/3,958	1.09 (1.04,1.16)	**$1.28x10^{-3}$**	0.049	3,441/912	1.07 (1.01,1.13)	0.114
<60 years	$4.70x10^{-3}$	1,607/6,457	1.12 (1.06,1.19)	**$3.44x10^{-5}$**	0.069	1,607/2,567	1.12 (1.05,1.20)	**$1.07x10^{-3}$**
<60 years Males	$4.70x10^{-3}$	987/2,904	1.16 (1.08,1.25)	**$6.39x10^{-5}$**	$7.85x10^{-3}$	987/649	1.12 (1.02,1.24)	0.020

#Results for the categories where the best-fit PRSice PRS showed a significant association with COVID-19 hospitalisation after multiple tests correction in the discovery stage. P-values showing a significant association after Bonferroni correction (4 tests, p≤0.013) are highlighted in bold. Association results for the best prediction model resulting from PRSice-2. PT: Best fit P-value Threshold. *A total of 281 individuals had missing data for sex (n=4) and age (n=277).

Table S5. Association of IPF whole-genome PRS with 90-day COVID-19 mortality

COVID-19 mortality category	Covariates	All individuals (Nt: 758 cases and 7,508 controls)			Excluding chronic respiratory diseases (Nt: 649 cases and 6,797 controls) †		
		PT	OR (95% CI)	P-value	PT	OR (95% CI)	P-value
All	Age, Sex, 10PC	$1.70x10^{-3}$	1.11 (1.02,1.20)	0.012	$1.70x10^{-3}$	1.11 (1.01,1.21)	0.023
Males	Age, 10PC	$8.5x10^{-4}$	1.14 (1.03,1.27)	0.014	$1.00x10^{-4}$	1.16 (1.03,1.31)	0.012
Females	Age, 10PC	$5.0x10^{-4}$	1.10 (0.96,1.25)	0.157	$4.3x10^{-3}$	1.07 (0.93,1.22)	0.336
≥60 years	Sex, 10PC	$9.5x10^{-4}$	1.09 (1.01,1.18)	0.033	$9.5x10^{-4}$	1.08 (0.99,1.18)	0.081
<60 years	Sex, 10PC	$4.5x10^{-4}$	1.27 (0.99,1.65)	0.063	0.011	1.27 (0.97,1.66)	0.083
≥60 years Males	10PC	$1.00x10^{-3}$	1.13 (1.01,1.26)	0.028	$1.00x10^{-4}$	1.16 (1.02,1.30)	0.019
≥60 years Females	10PC	0.416	0.91 (0.80,1.03)	0.149	0.351	0.90 (0.79,1.03)	0.121
<60 years Males	10PC	0.460	0.75 (0.53,1.06)	0.108	0.460	0.74 (0.51,1.06)	0.097
<60 years Females	10PC	0.011	1.55 (1.03,2.34)	0.035	0.011	1.64 (1.07,2.53)	0.024

PRSice-2 association results for the best prediction model in each category. †A total of 820 patients with chronic respiratory diseases were excluded. Nt: total sample size; PC: Principal Components; PT: Best fit P-value Threshold. Significance declared at $p<5.6x10^{-3}$ after Bonferroni correction (0.05/9 tests).

Table S6. Association with COVID-19 hospitalisation after excluding the *MUC5B* locus

			Whole-genome PRS			Sentinels PRS†	
COVID-19 hospitalisation category#	Covariates	*MUC5B* included	PT	OR (95% CI)	P-value*	OR (95%CI)	P-value
All	Age, Sex, 10PC	Yes	2.10×10^{-3}	1.08 (1.04,1.12)	**7.90×10^{-5}**	1.01 (0.97,1.05)	0.772
		No	2.10×10^{-3}	1.09 (1.05,1.14)	**5.55×10^{-6}**	1.06 (1.02,1.10)	**4.97×10^{-3}**
Males	Age, 10PC	Yes	7.85×10^{-3}	1.09 (1.04,1.16)	**1.28×10^{-3}**	1.05 (0.99,1.10)	0.107
		No	7.85×10^{-3}	1.10 (1.04,1.17)	**4.39×10^{-4}**	1.12 (1.06,1.18)	**7.55×10^{-5}**
<60 years	Sex, 10PC	Yes	4.70×10^{-3}	1.12 (1.06,1.19)	**3.44×10^{-5}**	1.02 (0.97,1.08)	0.415
		No	4.70×10^{-3}	1.14 (1.08,1.21)	**3.11×10^{-6}**	1.10 (1.04,1.16)	**9.34×10^{-4}**
<60 years Males	10PC	Yes	4.70×10^{-3}	1.16 (1.08,1.25)	**6.39×10^{-5}**	1.08 (1.00,1.16)	0.039
		No	4.70×10^{-3}	1.19 (1.10,1.28)	**5.56×10^{-6}**	1.19 (1.10,1.28)	**3.67×10^{-6}**

#Results for the categories where the best-fit PRSice PRS showed a significant association with COVID-19 hospitalisation after multiple tests correction in the discovery stage. P-values showing a significant association after Bonferroni correction (8 tests, $p \leq 6.25 \times 10^{-3}$) are highlighted in bold. To assess the PRS models after excluding *MUC5B*, the *MUC5B* promoter variant rs35705950 and variants within ±1 Mb were removed from the analyses. PC: Principal Components; PT: Best fit P-value Threshold. *PRSice-2 association results for the best prediction model in each category. †Association results for PRS calculated using the 19 previously reported genome-wide significant IPF common risk variants shown in Table S1. No thresholding was applied to sentinels PRS analyses.

Table S7. Results from the pathway-specific PRS analyses

Pathway [Number of SNPs included in the PRS*]	COVID-19 hospitalisation category#	Co-variables	OR (95%CI)	P-value
Cadherin pathway [69]	All	Age, Sex, 10PC	1.03 (0.99, 1.07)	0.171
	Males	Age, 10PC	1.06 (1.01, 1.12)	**0.028**
	<60 years	Sex, 10PC	1.03 (0.98, 1.09)	0.249
	<60 years Males	10PC	1.07 (1.00, 1.15)	0.057
Wnt pathway [102]	All	Age, Sex, 10PC	1.01 (0.97, 1.05)	0.531
	Males	Age, 10PC	1.02 (0.96, 1.07)	0.584
	<60 years	Sex, 10PC	1.02 (0.96, 1.08)	0.524
	<60 years Males	10PC	1.02 (0.95, 1.09)	0.649
Integrin pathway [73]	All	Age, Sex, 10PC	1.03 (0.99, 1.07)	0.103
	Males	Age, 10PC	1.03 (0.97, 1.08)	0.352
	<60 years	Sex, 10PC	1.08 (1.02, 1.14)	**0.007**
	<60 years Males	10PC	1.07 (0.99, 1.15)	0.078
Heterotrimeric G-protein signaling pathway-Gq alpha and Go alpha mediated [50]	All	Age, Sex, 10PC	1.00 (0.96, 1.04)	0.872
	Males	Age, 10PC	0.97 (0.91, 1.02)	0.214
	<60 years	Sex, 10PC	1.00 (0.95, 1.06)	0.907
	<60 years Males	10PC	0.99 (0.92, 1.06)	0.737
Heterotrimeric G-protein signaling pathway-Gi alpha and Gs alpha mediated [60]	All	Age, Sex, 10PC	1.01 (0.97, 1.05)	0.642
	Males	Age, 10PC	0.99 (0.93, 1.04)	0.352
	<60 years	Sex, 10PC	1.03 (0.97, 1.09)	0.320
	<60 years Males	10PC	0.99 (0.92, 1.07)	0.844

*SNPs reaching a P-value Threshold of 2.1×10^{-3} (i.e., the best fit P-value Threshold when considering all hospitalised individuals). #Results for the categories where the best-fit PRSice PRS showed a significant association with COVID-19 hospitalisation after multiple tests correction in the discovery stage. PC: Principal Components; SNP: single nucleotide polymorphism. Significance declared at $p=2.1 \times 10^{-3}$ after Bonferroni correction (20 tests). P-values showing a nominal association are highlighted in bold.

Supplementary Figures

Figure S1. Quartile plots of COVID-19 severity PRS

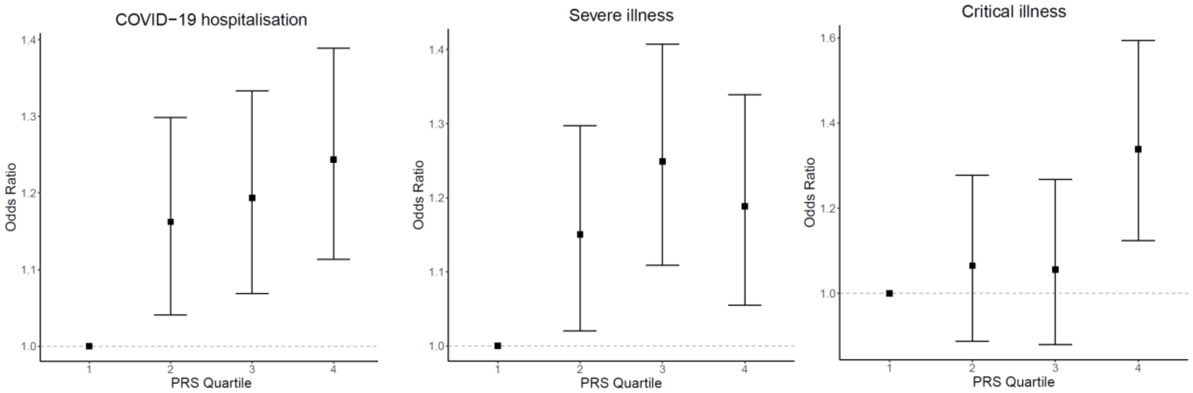

Figure S2. Forest plots of association results between IPF PRS and COVID-19 Hospitalisation (top), Severe illness (middle), and Critical illness (bottom).

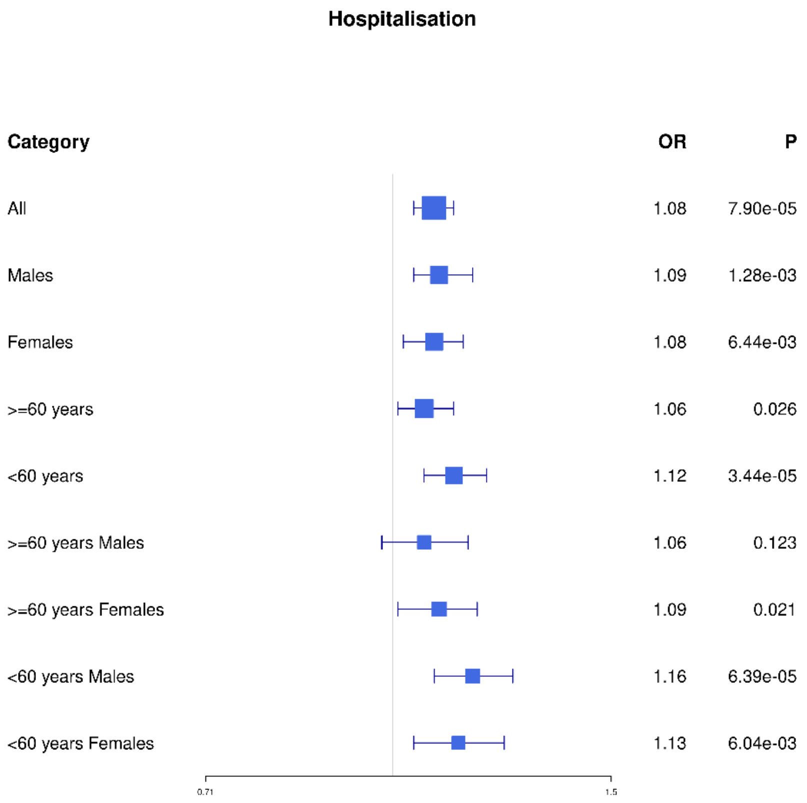

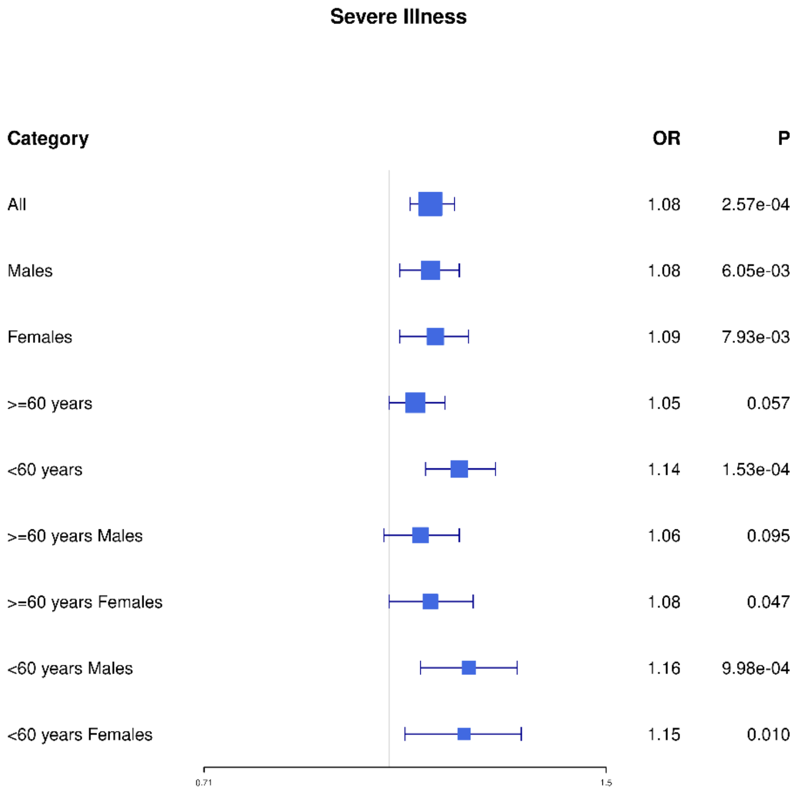

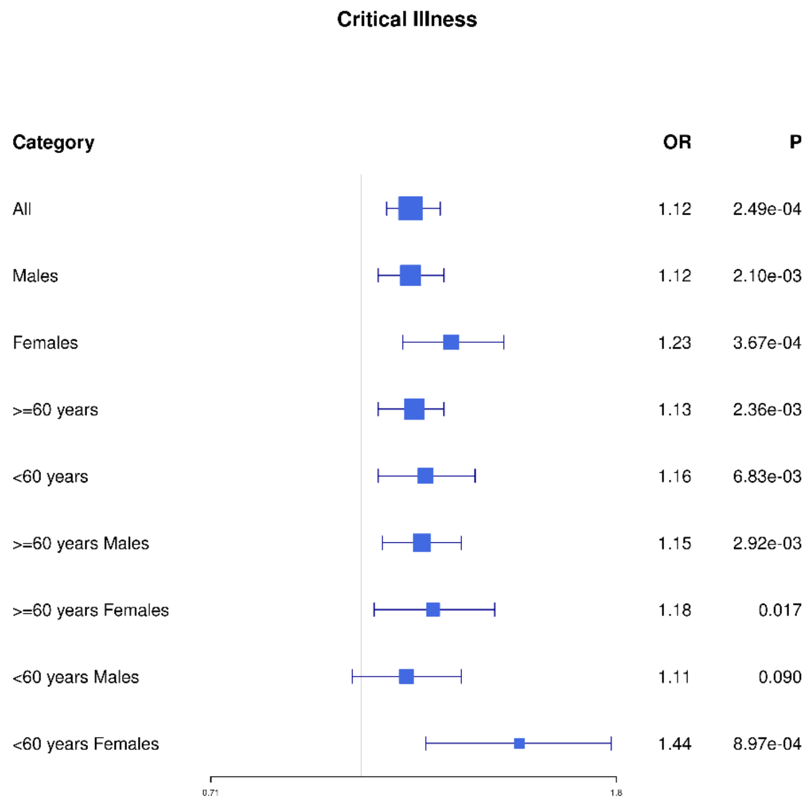

Figure S3. High-resolution PRSice-2 plot for the association of IPF PRS with COVID-19 hospitalisation. The best-fit PRS is obtained at P-value Threshold (PT) of 2.10x10^{-3}, with a whole-genome PRS model including 2,939 variants from the GWAS of IPF dataset.

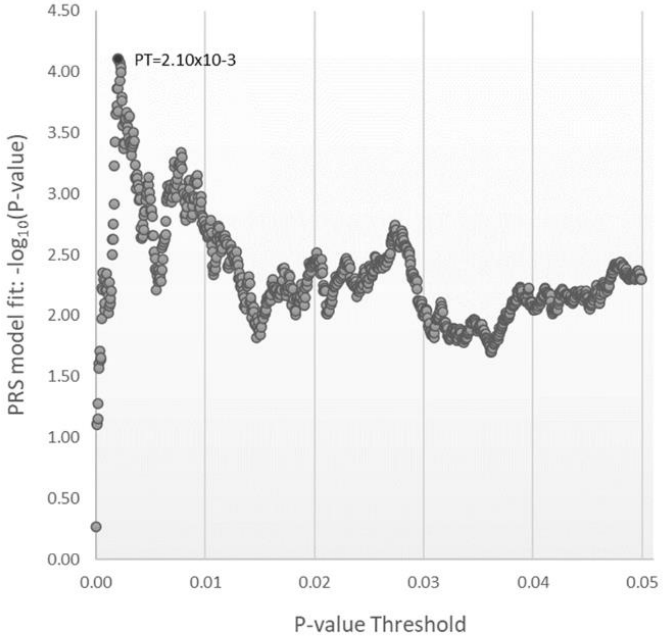

Figure S4. Pathway enrichment analysis for genetic factors included in the best-fit model of association of IPF whole-genome PRS with COVID-19 hospitalisation for all individuals. A) Analyses including all 2,939 SNPs used to calculate the PRS and their 3,471 mapping genes. B) Analyses excluding the 19 previously reported IPF common loci (sentinels ±1MB) (2,709 SNPs and 3,291 mapping genes left). All analyses were performed with ShinyGO v0.77 [5]. Top five biological pathways according to PANTHER database are shown.

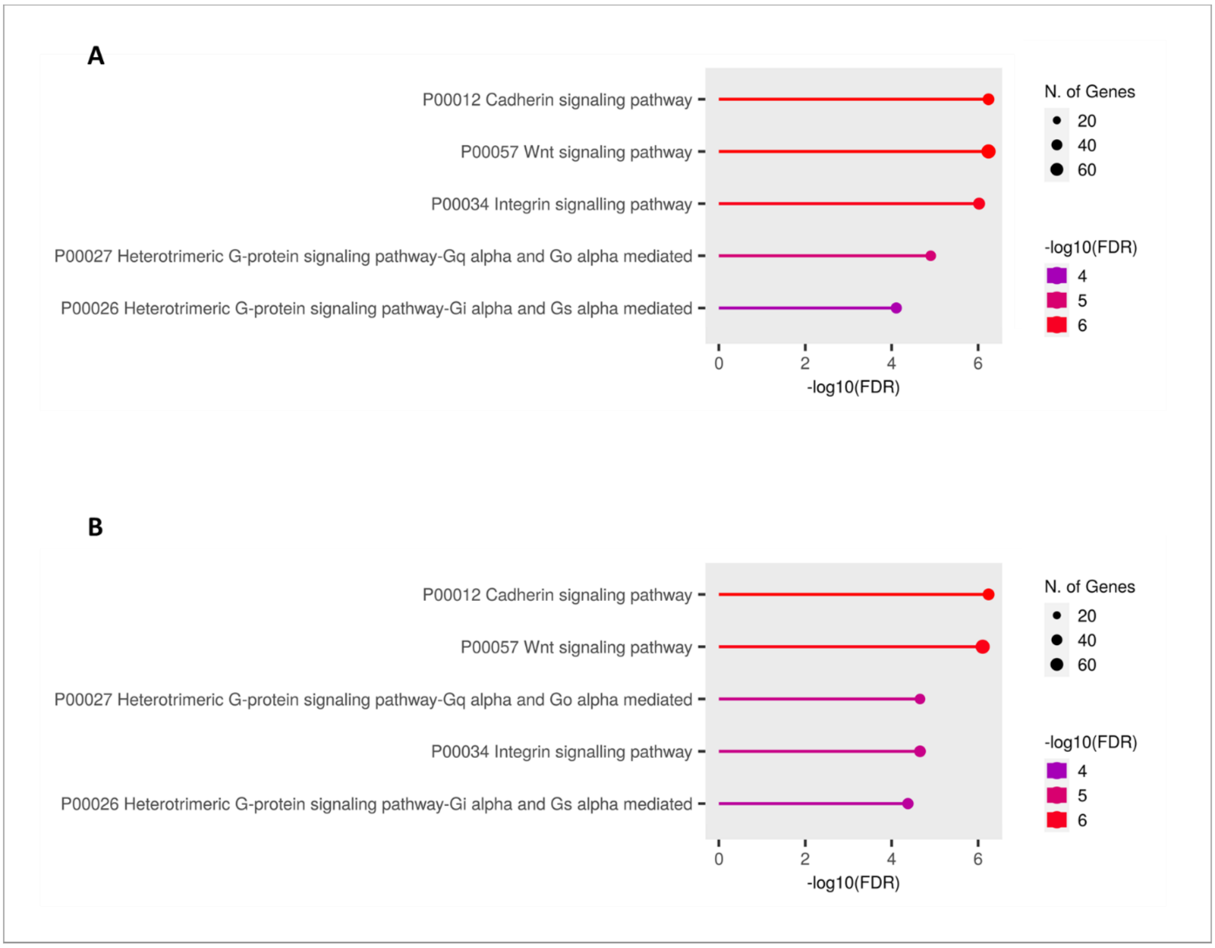

Figure S5. Pathway enrichment analysis for genetic factors included in the best-fit model of association of IPF whole-genome PRS with COVID-19 hospitalisation for <60 years males. Analyses including 5,660 SNPs used to calculate the PRS and their 5,424 mapping genes. Analyses were performed with ShinyGO v0.77 [5]. Top five biological pathways according to PANTHER database are shown.

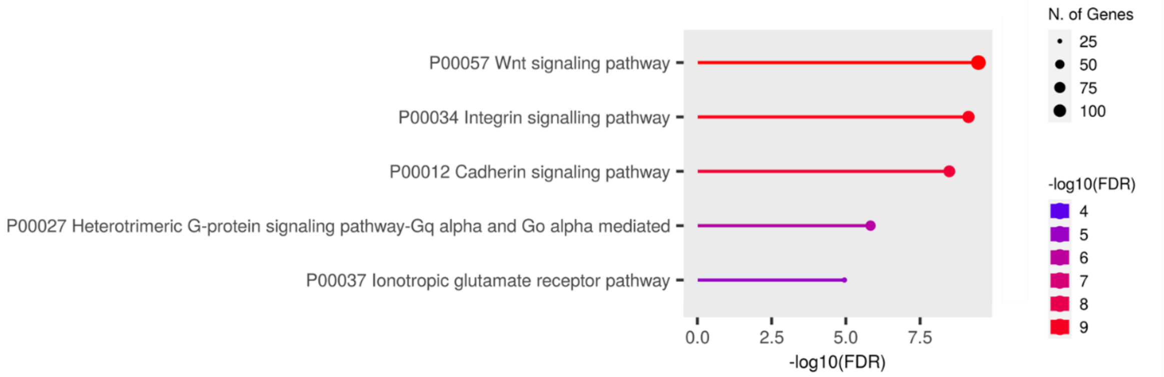

References

1. Harris PA, Taylor R, Thielke R, Payne J, Gonzalez N, Conde JG: Research electronic data capture (REDCap)--a metadata-driven methodology and workflow process for providing translational research informatics support. *J Biomed Inform* 2009, 42(2):377-381.
2. Harris PA, Taylor R, Minor BL, Elliott V, Fernandez M, O'Neal L, McLeod L, Delacqua G, Delacqua F, Kirby J *et al*: The REDCap consortium: Building an international community of software platform partners. *J Biomed Inform* 2019, 95:103208.
3. Allen RJ, Stockwell A, Oldham JM, Guillen-Guio B, Schwartz DA, Maher TM, Flores C, Noth I, Yaspan BL, Jenkins RG *et al*: Genome-wide association study across five cohorts identifies five novel loci associated with idiopathic pulmonary fibrosis. *Thorax* 2022, 77(8):829-833.
4. Dhindsa RS, Mattsson J, Nag A, Wang Q, Wain LV, Allen R, Wigmore EM, Ibanez K, Vitsios D, Deevi SVV *et al*: Identification of a missense variant in SPDL1 associated with idiopathic pulmonary fibrosis. *Commun Biol* 2021, 4(1):392.
5. Ge SX, Jung D, Yao R: ShinyGO: a graphical gene-set enrichment tool for animals and plants. *Bioinformatics* 2020, 36(8):2628-2629.

SCOURGE Cohort Group Members

Javier Abellan[1,2]; René Acosta-Isaac[3]; Jose María Aguado[4,5,6,7]; Carlos Aguilar[8]; Sergio Aguilera-Albesa[9,10]; Abdolah Ahmadi Sabbagh[11]; Jorge Alba[12]; Sergiu Albu[13,14,15]; Karla A.M. Alcalá-Gallardo[16]; Julia Alcoba-Florez[17]; Sergio Alcolea Batres[18]; Holmes Rafael Algarin-Lara[19,20]; Virginia Almadana[21]; Julia Almeida[22,23,24,25]; Berta Almoguera[26,27]; María R. Alonso[28]; Nuria Alvarez[28]; Yady Álvarez-Benítez[19,20]; Felipe Álvarez-Navia[29,30]; Rodolfo Alvarez-Sala Walther[18]; Álvaro Andreu-Bernabeu[31,6]; Maria Rosa Antonijoan[32]; Eunate Arana-Arri[33,34]; Carlos Aranda[35,36]; Celso Arango[31,37,6]; Carolina Araque[38,39]; Nathalia K. Araujo[40]; Izabel M.T. Araujo[41]; Ana C. Arcanjo[42,43,44]; Ana Arnaiz[45,46,47]; Francisco Arnalich Fernández[48]; María J. Arranz[49]; José Ramon Arribas Lopez[48]; Maria-Jesus Artiga[50]; Yubelly Avello-Malaver[51]; Carmen Ayuso[26,27]; Ana María Baldion[51]; Belén Ballina Martín[11]; Raúl C. Baptista-Rosas[52,53,54]; Andrea Barranco-Díaz[20]; María Barreda- Sánchez[55,56]; Viviana Barrera-Penagos[51]; Moncef Belhassen-Garcia[57,30]; Enrique Bernal[55]; David Bernal-Bello[58]; Joao F. Bezerra[59]; Marcos A.C. Bezerra[60]; Natalia Blanca-López[61]; Rafael Blancas[62]; Lucía Boix-Palop[63]; Alberto Borobia[64]; Elsa Bravo[65]; María Brion[66,67]; Óscar Brochado-Kith[68,7]; Ramón Brugada[69,70,67,71]; Matilde Bustos[72]; Alfonso Cabello[73]; Juan J. Caceres-Agra[74]; Esther Calbo[75]; Enrique J. Calderón[76,77,78]; Shirley Camacho[79]; Marcela C. Campos[42]; Yolanda Cañadas[36]; Cristina Carbonell[29,30]; Servando Cardona-Huerta[80]; Antonio Augusto F. Carioca[81]; Maria Sanchez Carpintero[35,36]; Carlos Carpio Segura[18]; Thássia M.T. Carratto[82]; José Antonio Carrillo-Avila[83]; Maria C.C. Carvalho[84]; Carlos Casasnovas[85,86,27]; Luis Castano[33,87,27,88,89]; Carlos F. Castaño[35,36]; Jose E. Castelao[90]; Aranzazu Castellano Candalija[91]; María A. Castillo[79]; Francisco C. Ceballos[68]; Jessica G. Chaux[39]; Walter G. Chaves- Santiago[92,39]; Sylena Chiquillo-Gómez[19,20]; Marco A. Cid-Lopez[16]; Oscar Cienfuegos-Jimenez[80]; Rosa Conde-Vicente[93]; M. Lourdes Cordero-Lorenzana[94]; Dolores Corella[95,96]; Almudena Corrales[97,98]; Jose L. Cortes-Sanchez[80,99]; Marta Corton[26,27]; Tatiana X. Costa[100]; Raquel Cruz[101,27,102,103]; Marina S. Cruz[40]; Luisa Cuesta[104]; Gabriela C.R. Cunha[105]; Gabriela V. da Silva[41]; David Dalmau[106,75]; Raquel C.S. Dantas-Komatsu[40]; M. Teresa Darnaude[107]; Raimundo de Andrés[108]; Jéssica N.G. de Araújo[109]; Carmen de Juan[110]; Juan De la Cruz Troca[111,112,77]; Carmen de la Horra[78]; Ana B. de la Hoz[33]; Alba De Martino-Rodríguez[113,114]; Julianna Lys de Sousa Alves Neri[115]; Victor del Campo-Pérez[116]; Juan Delgado-Cuesta[117]; Covadonga M. Diaz-Caneja[31,37,6]; Anderson Díaz-Pérez[20]; Aranzazu Diaz de Bustamante[107]; Beatriz Dietl[75]; Silvia Diz-de Almeida[27,103]; Manoella do Monte Alves[118,119]; Elena Domínguez-Garrido[120]; Katiusse A. dos Santos[84]; Alice M. Duarte[41]; Jose Echave-Sustaeta[121]; Rocío Eiros[122]; César O. Enciso-Olivera[38,39]; Gabriela Escudero[123]; Pedro Pablo España[124]; Gladys Mercedes Estigarribia Sanabria[125]; María Carmen Fariñas[45,46,47]; Marianne R. Fernandes[126,127]; Ramón Fernández[45,128]; Lidia Fernandez-Caballero[26,27]; Ana Fernández-Cruz[129]; María J. Fernandez-Nestosa[130]; Uxía Fernández-Robelo[131]; Amanda Fernández-Rodríguez[68,7]; Marta Fernández-Sampedro[45,47,46]; Ruth Fernández-Sánchez[26,27]; Tania Fernández-Villa[132]; Silvia Fernández Ferrero[11]; Yolanda Fernández Martínez[11]; Carmen Fernéndez Capitán[91]; Patricia Flores-Pérez[133]; Vicente Friaza[77,78]; Lácides Fuenmayor-Hernández[20]; Marta Fuertes Núñez[11]; Victoria Fumadó[134]; Ignacio Gadea[135]; Lidia Gagliardi[35,36]; Manuela Gago-Domínguez[136,102]; Natalia Gallego[137]; Cristina Galoppo[138]; Inés García[26,27]; Mercedes García[35,36]; Leticia García[35,36]; Carlos Garcia-Cerrada[1,2,139]; Aitor García-de-Vicuña[33,140]; Josefina Garcia-García[55]; Irene García-García[64]; Carmen García-Ibarbia[45,47,46]; Andrés C. García-Montero[141]; Ana García-Soidán[142]; Elisa García-Vázquez[55]; María Carmen García Torrejón[143,2]; Emiliano Garza-Frias[80]; Angela Gentile[138]; Belén Gil-Fournier[144]; Javier Gómez-Arrue[113,114]; Mario Gómez-Duque[92,39]; Luis Gómez Carrera[18]; María Gómez García[101]; Ángela Gómez Sacristán[145]; Anna González-Neira[28]; Javier González-Peñas[31,6,37]; Manuel Gonzalez-Sagrado[93]; Beatriz González Álvarez[113,114]; Fernan Gonzalez Bernaldo de Quirós[146]; Hugo Gonzalo Benito[147]; Oscar Gorgojo-Galindo[148]; Miguel Górgolas[73]; Florencia Guaragna[138]; Genilson P. Guegel[149]; Beatriz Guillen-Guio[97]; Encarna Guillen-Navarro[55,150,151,152]; Pablo Guisado-Vasco[121]; Juan F. Gutiérrez-Bautista[153]; Luz D. Gutierrez-Castañeda[154,39]; Sarah Heili-Frades[155]; Estefania Hernandez[156]; Luis D. Hernandez-Ortega[157,158]; Guillermo Hernández-Pérez[29]; Rebeca Hernández-Vaquero[159]; Cristina Hernández Moro[11]; Belen Herraez[28]; M. Teresa Herranz[55]; María Herrera[35,36]; María José Herrero[160,161]; Antonio Herrero-Gonzalez[162]; Juan P. Horcajada[163,164,14,165,7]; Natale Imaz-Ayo[33]; Maider Intxausti-Urrutibeaskoa[166]; María Íñiguez[167]; Rafael H. Jacomo[168]; Rubén Jara[55]; Perez Maria Jazmin[138]; Ángel Jiménez[35,36]; Pilar Jiménez[153]; Ignacio Jiménez-Alfaro[169]; María A. Jimenez-Sousa[68,7]; Iolanda Jordan[170,171,77]; Rocío Laguna-Goya[172,173]; Daniel Laorden[18]; María Lasa-Lazaro[172,173]; María Claudia Lattig[79,174]; Ailen Lauriente[138]; Anabel Liger Borja[175]; Lucía Llanos[176]; Amparo López-Bernús[29,30]; Esther Lopez-Garcia[111,112,77,177]; Rosario Lopez-Rodriguez[26,27]; Miguel A. López-Ruz[178,179,180]; Eduardo López Granados[181,182,27]; Leonardo Lorente[183]; José E. Lozano[184]; María Lozano-Espinosa[175]; Andre D. Luchessi[185]; Ignacio Mahillo[186,187,98]; Esther Mancebo[172,173]; Carmen Mar[124]; Cristina Marcelo Calvo[91]; Miguel Marcos[29,30]; Alba Marcos-Delgado[188]; Alicia Marín Candon[64]; Pablo Mariscal Aguilar[18]; María M. Martín[189]; María Dolores Martín[190]; Vicente Martín[188,77]; Marta Martin-Fernandez[191]; Caridad Martín-López[175]; José-Ángel Martín-Oterino[29,30]; Laura Martin-Pedraza[61]; María Martín-Vicente[68]; Amalia Martinez[192]; Ricardo Martínez[156]; Juan José Martínez[86,27]; Silvia Martínez[45,47]; Eleno Martínez-Aquino[193]; Óscar Martínez-González[194]; Iciar Martinez-Lopez[195,196]; Oscar Martinez-Nieto[51,174]; Pedro Martinez-Paz[147]; Angel Martinez-

Perez[197]; Andrea Martínez-Ramas[26,27]; Michel F. Martinez-Resendez[80]; Violeta Martínez Robles[11]; Laura Marzal[26,27]; Juliana F. Mazzeu[198,199,200]; Jeane F.P. Medeiros[40]; Kelliane A. Medeiros[201,202]; Francisco J. Medrano[76,77,78]; Xose M. Meijome[203,204]; Natalia Mejuto-Montero[205]; Ana Méndez-Echevarria[206]; Humberto Mendoza Charris[65,20]; Eleuterio Merayo Macías[207]; Fátima Mercadillo[208]; Arieh R. Mercado-Sesma[157,158]; Pablo Minguez[26,27]; Antonio J J. Molina[188,77]; Elena Molina-Roldán[209]; Juan José Montoya[156]; Vitor M.S. Moraes[82]; Patricia Moreira-Escriche[110]; Xenia Morelos-Arnedo[65,20]; Antonio Moreno-Docón[55]; Junior Moreno-Escalante[20]; Victor Moreno Cuerda[1,2]; Alberto Moreno Fernández[91]; Rubén Morilla[78,210]; Patricia Muñoz García[211,98,6]; Pablo Neira[138]; Julian Nevado[27,137,212]; Israel Nieto-Gañán[142]; Joana F.R. Nunes[42]; Rocio Nuñez- Torres[28]; Antònia Obrador-Hevia[213,214]; J. Gonzalo Ocejo-Vinyals[45,47]; Virginia Olivar[138]; Silviene F. Oliveira[198,215,216,217,218]; Lorena Ondo[26,27]; Alberto Orfao[22,23,24,25]; Luis Ortega[219]; Eva Ortega-Paino[50]; Fernando Ortiz-Flores[45,47]; Rocio Ortiz-Lopez[220,80]; José A. Oteo[12,167]; Harry Pachajoa[221,222]; Manuel Pacheco[156]; Fredy Javier Pacheco-Miranda[20]; Irene Padilla Conejo[11]; Sonia Panadero-Fajardo[83]; Mara Parellada[31,37,6]; Roberto Pariente-Rodríguez[142]; Estela Paz-Artal[172,173,223]; Germán Peces-Barba[224,98]; Miguel S. Pedromingo Kus[225]; Celia Perales[135]; Patricia Perez[226]; César Pérez[227]; Gustavo Perez-de-Nanclares[33,87]; Felipe Pérez-García[228,229]; Patricia Pérez-Matute[167]; Alexandra Pérez-Serra[69,67]; M. Elena Pérez-Tomás[55]; Teresa Perucho[230]; Lisbeth A. Pichardo[11]; Susana M.T. Pinho[201,231,232]; Mel·lina Pinsach-Abuin[69,67]; Luz Adriana Pinzón[92,39]; Guillermo Pita[28]; Francesc Pla-Junca[233,27]; Laura Planas-Serra[86,27]; Ericka N. Pompa-Mera[234]; Gloria L. Porras-Hurtado[156]; Aurora Pujol[86,27,235]; María Eugenia Quevedo Chávez[19,20]; Maria Angeles Quijada[32,236]; Inés Quintela[101]; Diana Ramirez-Montaño[237]; Soraya Ramiro León[144]; Pedro Rascado Sedes[238]; Delia Recalde[113,114]; Emma Recio-Fernández[167]; Salvador Resino[68,7]; Adriana P. Ribeiro[201,202,232]; Carlos S. Rivadeneira-Chamorro[39]; Diana Roa-Agudelo[51]; Montserrat Robelo Pardo[238]; Marilyn Johanna Rodriguez[39]; Fernando Rodriguez-Artalejo[111,112,77,177]; Marena Rodríguez-Ferrer[20]; Carlos Rodriguez-Gallego[239,240]; José A. Rodriguez-Garcia[11]; María A. Rodriguez-Hernandez[72]; Antonio Rodriguez-Nicolas[153]; Agustí Rodriguez-Palmero[241,86]; Emilio Rodríguez-Ruiz[238,102]; Paula A. Rodriguez-Urrego[51]; Belén Rodríguez Maya[1]; German Ezequiel Rodriguez Novoa[138]; Federico Rojo[242,25]; Andrea Romero-Coronado[20]; Filomeno Rondón García[11]; Lidia S. Rosa[243]; Antonio Rosales-Castillo[244]; Cladelis Rubio[245,246]; María Rubio Olivera[35,36]; Montserrat Ruiz[86,27]; Francisco Ruiz-Cabello[153,179,247]; Eva Ruiz-Casares[230]; Juan J. Ruiz-Cubillan[45,47]; Javier Ruiz-Hornillos[248,36,249]; Pablo Ryan[250,251,252]; Hector D. Salamanca[38,39]; Lorena Salazar-García[79]; Giorgina Gabriela Salgueiro Origlia [91]; Pedro-Luis Sánchez[122,30]; Clara Sánchez-Pablo[122]; Olga Sánchez-Pernaute[253]; Antonio J. Sánchez López[254]; María Concepción Sánchez Prados[18]; Javier Sánchez Real[11]; Jorge Sánchez Redondo[1,255]; Cristina Sancho- Sainz[166]; Anna Sangil[63]; Arnoldo Santos[227]; Ney P.C. Santos[126]; Agatha Schlüter[86,27]; Sonia Segovia[233,256,257]; Alex Serra-Llovich[258]; Fernando Sevil Puras[8]; Marta Sevilla Porras[27,137]; Miguel A. Sicolo[259,260]; Vivian N. Silbiger[185]; Nayara S. Silva[109]; Fabiola T.C. Silva[42]; Cristina Silván Fuentes[27]; Jordi Solé-Violán[261,98,262]; José Manuel Soria[197]; Jose V. Sorlí[95,96]; Renata R. Sousa[198]; Juan Carlos Souto[3]; Karla S.C. Souza[84]; Vanessa S. Souza[105]; John J. Sprockel[92,39]; José Javier Suárez-Rama[101]; David A. Suarez-Zamora[51]; Xiana Taboada-Fraga[205]; Eduardo Tamayo[263,148]; Alvaro Tamayo-Velasco[264]; Juan Carlos Taracido-Fernandez[162]; Nathali A.C. Tavares[265]; Carlos Tellería[113,114]; Jair Antonio Tenorio Castaño[27,137,212]; Alejandro Teper[138]; Javier Torres[234]; Juan Torres-Macho[266]; Lilian Torres-Tobar[39]; Ronald P. Torres Gutiérrez[225]; Jesús Troya[250]; Miguel Urioste[208]; Juan Valencia-Ramos[267]; Agustín Valido[21,268]; Juan Pablo Vargas Gallo[269,270]; Belén Varón[271]; Romero H.T. Vasconcelos[265]; Tomas Vega[272]; Santiago Velasco-Quirce[273]; Valentina Vélez-Santamaría[85,86]; Virginia Víctor[35,36]; Julia Vidán Estévez[11]; Miriam Vieitez-Santiago[45,47]; Carlos Vilches[274]; Lavinia Villalobos[11]; Felipe Villar[224]; Judit Villar-Garcia[275,276,277]; Cristina Villaverde[26,27]; Pablo Villoslada-Blanco[167]; Ana Virseda-Berdices[68]; Zuleima Yáñez[20]; Antonio Zapatero-Gaviria[278]; Ruth Zarate[279]; Sandra Zazo[242]; Alba Espinoza-Altamirano[280]; Jesús Gaytán-Martínez[280]; Juan Carlos Hermosillo-Domínguez[280]; José Antonio Mata-Marín[280]; Mireya Núñez-Armendáriz[280]; Jorge Luis Sandoval-Ramírez[280]; Miguel López de Heredia[27]; Ingrid Mendes[27]; Rocío Moreno[27]; Esther Sande[27,102,103]; Carlos Flores[281,97,98,240]; José A. Riancho[45,46,47]; Augusto Rojas-Martinez[80]; Pablo Lapunzina[27,137,212]; Angel Carracedo[101,27,102,103,136]

SCOURGE Cohort Group Affiliations

[1], Hospital Universitario Mostoles, Medicina Interna, Madrid, Spain
[2], Universidad Francisco de Vitoria, Madrid, Spain
[3], Haemostasis and Thrombosis Unit, Hospital de la Santa Creu i Sant Pau, IIB Sant Pau, Barcelona, Spain
[4], Unit of Infectious Diseases, Hospital Universitario 12 de Octubre, Instituto de Investigación Sanitaria Hospital 12 de Octubre (imas12), Madrid, Spain
[5], Spanish Network for Research in Infectious Diseases (REIPI RD16/0016/0002), Instituto de Salud Carlos III, Madrid, Spain
[6], School of Medicine, Universidad Complutense, Madrid, Spain
[7], Centro de Investigación Biomédica en Red de Enfermedades Infecciosas (CIBERINFEC), Instituto de Salud Carlos III, Madrid, Spain
[8], Hospital General Santa Bárbara de Soria, Soria, Spain

[9], Pediatric Neurology Unit, Department of Pediatrics, Navarra Health Service Hospital, Pamplona, Spain
[10], Navarra Health Service, NavarraBioMed Research Group, Pamplona, Spain
[11], Complejo Asistencial Universitario de León, León, Spain
[12], Hospital Universitario San Pedro, Infectious Diseases Department, Logroño, Spain
[13], Fundación Institut Guttmann, Institut Universitari de Neurorehabilitació adscrit a la UAB, Hospital de Neurorehabilitació, Barcelona, Spain
[14], Universitat Autònoma de Barcelona (UAB), Barcelona, Spain
[15], Fundació Institut d'Investigació en Ciències de la Salut Germans Trias i Pujol, Barcelona, Spain
[16], Hospital General de Occidente, Guadalajara, Mexico
[17], Microbiology Unit, Hospital Universitario N. S. de Candelaria, Santa Cruz de Tenerife, Spain
[18], Hospital Universitario La Paz-IDIPAZ, Servicio de Neumología, Madrid, Spain
[19], Camino Universitario Adelita de Char, Mired IPS, Barranquilla, Colombia
[20], Universidad Simón Bolívar, Facultad de Ciencias de la Salud, Barranquilla, Colombia
[21], Hospital Universitario Virgen Macarena, Neumología, Seville, Spain
[22], Departamento de Medicina, Universidad de Salamanca, Salamanca, Spain
[23], Centro de Investigación del Cáncer (IBMCC) Universidad de Salamanca - CSIC, Salamanca, Spain
[24], Biomedical Research Institute of Salamanca (IBSAL) Salamanca, Spain
[25], Centre for Biomedical Network Research on Cancer (CIBERONC), Instituto de Salud Carlos III, Madrid, Spain
[26], Department of Genetics & Genomics, Instituto de Investigación Sanitaria-Fundación Jiménez Díaz University Hospital - Universidad Autónoma de Madrid (IIS-FJD, UAM), Madrid, Spain
[27], Centre for Biomedical Network Research on Rare Diseases (CIBERER), Instituto de Salud Carlos III, Madrid, Spain
[28], Spanish National Cancer Research Centre, Human Genotyping-CEGEN Unit, Madrid, Spain
[29], Hospital Universitario de Salamanca-IBSAL, Servicio de Medicina Interna, Salamanca, Spain
[30], Universidad de Salamanca, Salamanca, Spain
[31], Department of Child and Adolescent Psychiatry, Institute of Psychiatry and Mental Health, Hospital General Universitario Gregorio Marañón (IiSGM), Madrid, Spain
[32], Clinical Pharmacology Service, Hospital de la Santa Creu i Sant Pau, IIB Sant Pau, Barcelona, Spain
[33], Biocruces Bizkai HRI, Barakaldo, Bizkaia, Spain
[34], Cruces University Hospital, Osakidetza, Barakaldo, Bizkaia, Spain
[35], Hospital Infanta Elena, Valdemoro, Madrid, Spain
[36], Instituto de Investigación Sanitaria-Fundación Jiménez Díaz University Hospital - Universidad Autónoma de Madrid (IIS-FJD, UAM), Madrid, Spain
[37], Centre for Biomedical Network Research on Mental Health (CIBERSAM), Instituto de Salud Carlos III, Madrid, Spain
[38], Fundación Hospital Infantil Universitario de San José, Bogotá, Colombia
[39], Fundación Universitaria de Ciencias de la Salud, Bogotá, Colombia
[40], Universidade Federal do Rio Grande do Norte, Programa de Pós-graduação em Ciências da Saúde, Natal, Brazil
[41], Universidade Federal do Rio Grande do Norte, Departamento de Medicina Clínica, Natal, Brazil
[42], Departamento de Genética e Morfologia, Instituto de Ciências Biológicas, Universidade de Brasília, Brasilia, Brazil
[43], Colégio Marista de Brasilia, Brazil
[44], Associação Brasileira de Educação e Cultura, Brazil
[45], IDIVAL, Santander, Spain
[46], Universidad de Cantabria, Santander, Spain
[47], Hospital U M Valdecilla, Santander, Spain
[48], Hospital Universitario La Paz-IDIPAZ, Servicio de Medicina Interna, Madrid, Spain
[49], Fundació Docència I Recerca Mutua Terrassa, Barcelona, Spain
[50], Spanish National Cancer Research Center, CNIO Biobank, Madrid, Spain
[51], Fundación Santa Fe de Bogota, Departamento Patologia y Laboratorios, Bogotá, Colombia
[52], Hospital General de Occidente, Zapopan, Jalisco, Mexico
[53], Centro Universitario de Tonalá, Universidad de Guadalajara, Tonalá, Jalisco, Mexico
[54], Centro de Investigación Multidisciplinario en Salud, Universidad de Guadalajara, Tonalá, Jalisco, Mexico
[55], Instituto Murciano de Investigación Biosanitaria (IMIB-Arrixaca), Murcia, Spain
[56], Universidad Católica San Antonio de Murcia (UCAM), Murcia, Spain
[57], Hospital Universitario de Salamanca-IBSAL, Servicio de Medicina Interna-Unidad de Enfermedades Infecciosas, Salamanca, Spain
[58], Hospital Universitario de Fuenlabrada, Department of Internal Medicine, Madrid, Spain
[59], Escola Tecnica de Saúde, Laboratorio de Vigilancia Molecular Aplicada, Pará, Brazil
[60], Federal University of Pernambuco, Genetics Postgraduate Program, Recife, PE, Brazil

[61], Hospital Universitario Infanta Leonor, Servicio de Alergia, Madrid, Spain
[62], Hospital Universitario del Tajo, Servicio de Medicina Intensiva, Aranjuez, Spain
[63], Hospital Universitario Mutua Terrassa, Barcelona, Spain
[64], Hospital Universitario La Paz-IDIPAZ, Servicio de Farmacología, Madrid, Spain
[65], Alcaldía de Barranquilla, Secretaría de Salud, Barranquilla, Colombia
[66], Instituto de Investigación Sanitaria de Santiago (IDIS), Xenética Cardiovascular, Santiago de Compostela, Spain
[67], Centre for Biomedical Network Research on Cardiovascular Diseases (CIBERCV), Instituto de Salud Carlos III, Madrid, Spain
[68], Unidad de Infección Viral e Inmunidad, Centro Nacional de Microbiología (CNM), Instituto de Salud Carlos III (ISCIII), Madrid, Spain
[69], Cardiovascular Genetics Center, Institut d'Investigació Biomèdica Girona (IDIBGI), Girona, Spain
[70], Medical Science Department, School of Medicine, University of Girona, Girona, Spain
[71], Hospital Josep Trueta, Cardiology Service, Girona, Spain
[72], Institute of Biomedicine of Seville (IBiS), Consejo Superior de Investigaciones Científicas (CSIC)- University of Seville- Virgen del Rocio University Hospital, Seville, Spain
[73], Division of Infectious Diseases, Instituto de Investigación Sanitaria-Fundación Jiménez Díaz University Hospital - Universidad Autónoma de Madrid (IIS-FJD, UAM), Madrid, Spain
[74], Intensive Care Unit, Hospital Universitario Insular de Gran Canaria, Las Palmas de Gran Canaria, Spain
[75], Hospital Universitario Mutua Terrassa, Terrassa, Spain
[76], Departemento de Medicina, Hospital Universitario Virgen del Rocío,Universidad de Sevilla, Seville, Spain
[77], Centre for Biomedical Network Research on Epidemiology and Public Health (CIBERESP), Instituto de Salud Carlos III, Madrid, Spain
[78], Instituto de Biomedicina de Sevilla, Seville, Spain
[79], Universidad de los Andes, Facultad de Ciencias, Bogotá, Colombia
[80], Tecnologico de Monterrey, Escuela de Medicina y Ciencias de la Salud and Hospital San Jose TecSalud, Monterrey, Mexico
[81], University of Fortaleza (UNIFOR), Department of Nutrition. Fortaleza, Brazil
[82], Departamento de Química, Faculdade de Filosofia, Ciências e Letras de Ribeirão Preto, Universidade de São Paulo, Brazil
[83], Andalusian Public Health System Biobank, Granada, Spain
[84], Universidade Federal do Rio Grande do Norte, Programa de Pós-Graduação em Ciências Farmacêuticas, Natal, Brazil
[85], Neuromuscular Unit, Neurology Department, Hospital Universitari de Bellvitge, L'Hospitalet de Llobregat (Barcelona), Spain
[86], Bellvitge Biomedical Research Institute (IDIBELL), Neurometabolic Diseases Laboratory, L'Hospitalet de Llobregat, Spain
[87], Osakidetza, Cruces University Hospital, Barakaldo, Bizkaia, Spain
[88], Centre for Biomedical Network Research on Diabetes and Metabolic Associated Diseases (CIBERDEM), Instituto de Salud Carlos III, Madrid, Spain
[89], University of Pais Vasco, UPV/EHU, Bizkaia, Spain
[90], Oncology and Genetics Unit, Instituto de Investigacion Sanitaria Galicia Sur, Xerencia de Xestion Integrada de Vigo-Servizo Galego de Saúde, Vigo, Spain
[91], Hospital Universitario La Paz, Hospital Carlos III, Madrid, Spain
[92], Hospital de San José, Sociedad de Cirugía de Bogota, Bogotá, Colombia
[93], Hospital Universitario Río Hortega, Valladolid, Spain
[94], Servicio de Medicina intensiva, Complejo Hospitalario Universitario de A Coruña (CHUAC), Sistema Galego de Saúde (SERGAS), A Coruña, Spain
[95], Valencia University, Preventive Medicine Department, Valencia, Spain
[96], Centre for Biomedical Network Research on Physiopatology of Obesity and Nutrition (CIBEROBN), Instituto de Salud Carlos III, Madrid, Spain
[97], Research Unit, Hospital Universitario N.S. de Candelaria, Santa Cruz de Tenerife, Spain
[98], Centre for Biomedical Network Research on Respiratory Diseases (CIBERES), Instituto de Salud Carlos III, Madrid, Spain
[99], Otto von Guericke University, Departament of Microgravity and Translational Regenerative Medicine, Magdeburg, Germany
[100], Maternidade Escola Janário Cicco, Natal, Brazil
[101], Centro Nacional de Genotipado (CEGEN), Universidade de Santiago de Compostela, Santiago de Compostela, Spain
[102], Instituto de Investigación Sanitaria de Santiago (IDIS), Santiago de Compostela, Spain

[103], Centro Singular de Investigación en Medicina Molecular y Enfermedades Crónicas (CIMUS), Universidade de Santiago de Compostela, Santiago de Compostela, Spain
[104], Institute of Psychiatry and Mental Health, Hospital General Universitario Gregorio Marañón (IiSGM), Madrid, Spain
[105], Programa de Pós Graduação em Ciências da Saúde, Faculdade de Medicina, Universidade de Brasília, Brasilia, Brazil
[106], Fundació Docència I Recerca Mutua Terrassa, Terrassa, Spain
[107], Hospital Universitario Mostoles, Unidad de Genética, Madrid, Spain
[108], Internal Medicine Department, Instituto de Investigación Sanitaria-Fundación Jiménez Díaz University Hospital - Universidad Autónoma de Madrid (IIS-FJD, UAM), Madrid, Spain
[109], Universidade Federal do Rio Grande do Norte, Pós-graduação em Biotecnologia - Rede de Biotecnologia do Nordeste (Renorbio), Natal, Brazil
[110], Hospital Universitario Severo Ochoa, Servicio de Medicina Interna, Madrid, Spain
[111], Department of Preventive Medicine and Public Health, School of Medicine, Universidad Autónoma de Madrid, Madrid, Spain
[112], IdiPaz (Instituto de Investigación Sanitaria Hospital Universitario La Paz), Madrid, Spain
[113], Instituto Aragonés de Ciencias de la Salud (IACS), Zaragoza, Spain
[114], Instituto Investigación Sanitaria Aragón (IIS-Aragon), Zaragoza, Spain
[115], Universidade Federal do Rio Grande do Norte, Programa de Pós Graduação em Nutrição, Natal, Brazil
[116], Preventive Medicine Department, Instituto de Investigacion Sanitaria Galicia Sur, Xerencia de Xestion Integrada de Vigo-Servizo Galego de Saúde, Vigo, Spain
[117], Hospital Universitario Virgen del Rocío, Servicio de Medicina Interna, Seville, Spain
[118], Universidade Federal do Rio Grande do Norte, Departamento de Infectologia, Natal, Brazil
[119], Hospital de Doenças Infecciosas Giselda Trigueiro, Rio Grande do Norte, Natal, Brazil
[120], Unidad Diagnóstico Molecular. Fundación Rioja Salud, La Rioja, Spain
[121], Hospital Universitario Quironsalud Madrid, Madrid, Spain
[122], Hospital Universitario de Salamanca-IBSAL, Servicio de Cardiología, Salamanca, Spain
[123], Hospital Universitario Puerta de Hierro, Servicio de Medicina Interna, Majadahonda, Spain
[124], Biocruces Bizkaia Health Research Institute, Galdakao University Hospital, Osakidetza, Bizkaia, Spain
[125], Instituto Regional de Investigación en Salud-Universidad Nacional de Caaguazú, Caaguazú, Paraguay
[126], Universidade Federal do Pará, Núcleo de Pesquisas em Oncologia, Belém, Pará, Brazil
[127], Hospital Ophir Loyola, Departamento de Ensino e Pesquisa, Belém, Pará, Brazil
[128], Fundación Asilo San Jose, Santander, Spain
[129], Unidad de Enfermedades Infecciosas, Servicio de Medicina Interna, Hospital Universitario Puerta de Hierro, Instituto de Investigación Sanitaria Puerta de Hierro - Segovia de Arana, Madrid, Spain
[130], Universidad Nacional de Asunción, Facultad de Politécnica, Paraguay
[131], Urgencias Hospitalarias, Complejo Hospitalario Universitario de A Coruña (CHUAC), Sistema Galego de Saúde (SERGAS), A Coruña, Spain
[132], Grupo de Investigación en Interacciones Gen-Ambiente y Salud (GIIGAS) - Instituto de Biomedicina (IBIOMED), Universidad de León, León, Spain
[133], Hospital Universitario Niño Jesús, Pediatrics Department, Madrid, Spain
[134], Unitat de Malalties Infeccioses i Importades, Servei de Pediatría, Infectious and Imported Diseases, Pediatric Unit, Hospital Universitari Sant Joan de Deú, Barcelona, Spain
[135], Microbiology Department, Instituto de Investigación Sanitaria-Fundación Jiménez Díaz University Hospital - Universidad Autónoma de Madrid (IIS-FJD, UAM), Madrid, Spain
[136], Fundación Pública Galega de Medicina Xenómica, Sistema Galego de Saúde (SERGAS) Santiago de Compostela, Spain
[137], Instituto de Genética Médica y Molecular (INGEMM), Hospital Universitario La Paz-IDIPAZ, Madrid, Spain
[138], Hospital de Niños Ricardo Gutierrez, Buenos Aires, Argentina
[139], Centre for Biomedical Network Research on Rare Diseases (CIBERER), Instituto de Salud Carlos III, Madrid, Spain Universidad Francisco de Vitoria, Madrid,Spain
[140], Osakidetza, Cruces University Hospital, Bizkaia, Spain
[141], University of Salamanca, Biomedical Research Institute of Salamanca (IBSAL), Salamanca, Spain
[142], Department of Immunology, IRYCIS, Hospital Universitario Ramón y Cajal, Madrid, Spain
[143], Hospital Infanta Elena, Servicio de Medicina Intensiva, Valdemoro, Madrid, Spain
[144], Hospital Universitario de Getafe, Servicio de Genética, Madrid, Spain
[145], Pneumology Department, Hospital General Universitario Gregorio Marañón (iiSGM), Madrid, Spain
[146], Ministerio de Salud Ciudad de Buenos Aires, Buenos Aires, Argentina
[147], Hospital Clinico Universitario de Valladolid, Unidad de Apoyo a la Investigación, Valladolid, Spain
[148], Universidad de Valladolid, Departamento de Cirugía, Valladolid, Spain

[149], Secretaria Municipal de Saude de Apodi, Natal, Brazil
[150], Sección Genética Médica - Servicio de Pediatría, Hospital Clínico Universitario Virgen de la Arrixaca, Servicio Murciano de Salud, Murcia, Spain
[151], Departamento Cirugía, Pediatría, Obstetricia y Ginecología, Facultad de Medicina, Universidad de Murcia (UMU), Murcia, Spain
[152], Grupo Clínico Vinculado, Centre for Biomedical Network Research on Rare Diseases (CIBERER), Instituto de Salud Carlos III, Madrid, Spain
[153], Hospital Universitario Virgen de las Nieves, Servicio de Análisis Clínicos e Inmunología, Granada, Spain
[154], Hospital Universitario Centro Dermatológico Federico Lleras Acosta, Bogotá, Colombia
[155], Intermediate Respiratory Care Unit, Department of Pneumology, Instituto de Investigación Sanitaria-Fundación Jiménez Díaz University Hospital - Universidad Autónoma de Madrid (IIS-FJD, UAM), Madrid, Spain
[156], Clinica Comfamiliar Risaralda, Pereira, Colombia
[157], Centro Universitario de Tonalá, Universidad de Guadalajara, Guadalajara, Mexico
[158], Centro de Investigación Multidisciplinario en Salud, Universidad de Guadalajara, Guadalajara, Mexico
[159], Unidad de Cuidados, Intensivos Hospital Clínico Universitario de Santiago (CHUS), Sistema Galego de Saúde (SERGAS), Santiago de Compostela, Spain
[160], IIS La Fe, Plataforma de Farmacogenética, Valencia, Spain
[161], Universidad de Valencia, Departamento de Farmacología, Valencia, Spain
[162], Data Analysis Department, Instituto de Investigación Sanitaria-Fundación Jiménez Díaz University Hospital - Universidad Autónoma de Madrid (IIS-FJD, UAM), Madrid, Spain
[163], Hospital del Mar, Infectious Diseases Service, Barcelona, Spain
[164], Institut Hospital del Mar d'Investigacions Mèdiques (IMIM), Barcelona, Spain
[165], CEXS-Universitat Pompeu Fabra, Spanish Network for Research in Infectious Diseases (REIPI), Barcelona, Spain
[166], Biocruces Bizkaia Health Research Institute, Basurto University Hospital, Osakidetza, Bizkaia, Spain
[167], Infectious Diseases, Microbiota and Metabolism Unit, Center for Biomedical Research of La Rioja (CIBIR), Logroño, Spain
[168], Sabin Medicina Diagnóstica, Brazil
[169], Opthalmology Department, Instituto de Investigación Sanitaria-Fundación Jiménez Díaz University Hospital - Universidad Autónoma de Madrid (IIS-FJD, UAM), Madrid, Spain
[170], Hospital Sant Joan de Deu,Pediatric Critical Care Unit, Barcelona, Spain
[171], Paediatric Intensive Care Unit, Agrupación Hospitalaria Clínic-Sant Joan de Déu, Esplugues de Llobregat, Barcelona, Spain
[172], Hospital Universitario 12 de Octubre, Department of Immunology, Madrid, Spain
[173], Instituto de Investigación Sanitaria Hospital 12 de Octubre (imas12), Transplant Immunology and Immunodeficiencies Group, Madrid, Spain
[174], SIGEN Alianza Universidad de los Andes - Fundación Santa Fe de Bogotá, Bogotá, Colombia
[175], Hospital General de Segovia, Medicina Intensiva, Segovia, Spain
[176], Clinical Trials Unit, Instituto de Investigación Sanitaria-Fundación Jiménez Díaz University Hospital - Universidad Autónoma de Madrid (IIS-FJD, UAM), Madrid, Spain
[177], IMDEA-Food Institute, CEI UAM+CSIC, Madrid, Spain
[178], Hospital Universitario Virgen de las Nieves, Servicio de Enfermedades Infecciosas, Granada, Spain
[179], Instituto de Investigación Biosanitaria de Granada (ibs GRANADA), Granada, Spain
[180], Universidad de Granada, Departamento de Medicina, Granada, Spain
[181], Hospital Universitario La Paz-IDIPAZ, Servicio de Inmunología, Madrid, Spain
[182], La Paz Institute for Health Research (IdiPAZ), Lymphocyte Pathophysiology in Immunodeficiencies Group, Madrid, Spain
[183], Intensive Care Unit, Hospital Universitario de Canarias, La Laguna, Spain
[184], Dirección General de Salud Pública, Consejería de Sanidad, Junta de Castilla y León, Valladolid, Spain
[185], Universidade Federal do Rio Grande do Norte, Departamento de Analises Clinicas e Toxicologicas, Natal, Brazil
[186], Fundación Jiménez Díaz, Epidemiology, Madrid, Spain
[187], Universidad Autónoma de Madrid, Department of Medicine, Madrid, Spain
[188], Instituto de Biomedicina (IBIOMED), Universidad de León, León, Spain
[189], Intensive Care Unit, Hospital Universitario N. S. de Candelaria, Santa Cruz de Tenerife, Spain
[190], Preventive Medicine Department, Instituto de Investigación Sanitaria-Fundación Jiménez Díaz University Hospital - Universidad Autónoma de Madrid (IIS-FJD, UAM), Madrid, Spain
[191], Universidad de Valladolid, Departamento de Medicina, Valladolid, Spain
[192], Hospital Universitario Infanta Leonor, Servicio de Medicina Intensiva, Madrid, Spain
[193], Servicio de Medicina Interna, Sanatorio Franchin, Buenos Aires, Argentina

[194], Hospital Universitario del Tajo, Servicio de Medicina Intensiva, Toledo, Spain
[195], Unidad de Genética y Genómica Islas Baleares, Islas Baleares, Spain
[196], Hospital Universitario Son Espases, Unidad de Diagnóstico Molecular y Genética Clínica, Islas Baleares, Spain
[197], Genomics of Complex Diseases Unit, Research Institute of Hospital de la Santa Creu i Sant Pau, IIB Sant Pau, Barcelona, Spain
[198], Faculdade de Medicina, Universidade de Brasília, Brasilia, Brazil
[199], Programa de Pós-Graduação em Ciências Médicas, Universidade de Brasília, Brasilia, Brazil
[200], Programa de Pós-Graduação em Ciências da Saúde, Universidade de Brasília, Brasilia, Brazil
[201], Hospital das Forças Armadas, Brazil
[202], Exército Brasileiro, Brazil
[203], Hospital El Bierzo, Gerencia de Asistencia Sanitaria del Bierzo (GASBI), Gerencia Regional de Salud (SACYL), Ponferrada, Spain
[204], Grupo INVESTEN, Instituto de Salud Carlos III, Madrid, Spain
[205], Unidad de Cuidados Intensivos, Complejo Universitario de A Coruña (CHUAC), Sistema Galego de Saúde (SERGAS), A Coruña, Spain
[206], Hospital Universitario La Paz-IDIPAZ, Servicio de Pediatría, Madrid, Spain
[207], Hospital El Bierzo, Unidad Cuidados Intensivos, León, Spain
[208], Spanish National Cancer Research Centre, Familial Cancer Clinical Unit, Madrid, Spain
[209], Instituto de Investigación Sanitaria San Carlos (IdISSC), Hospital Clínico San Carlos (HCSC), Madrid, Spain
[210], Universidad de Sevilla, Departamento de Enfermería, Seville, Spain
[211], Hospital General Universitario Gregorio Marañón (IiSGM), Madrid, Spain
[212], ERN-ITHACA-European Reference Network
[213], Unidad de Genética y Genómica Islas Baleares, Unidad de Diagnóstico Molecular y Genética Clínica, Hospital Universitario Son Espases, Islas Baleares, Spain
[214], Instituto de Investigación Sanitaria Islas Baleares (IdISBa), Islas Baleares, Spain
[215], Programa de Pós-Graduação em Biologia Animal, Universidade de Brasília, Brasília, Brazil
[216], Programa de Pós-Graduação em Ciências da Saúde, Universidade de Brasília, Brasília, Brazil
[217], Programa de Pós-Graduação Profissional em Ensino de Biologia, Universidade de Brasília, Brasília, Brazil
[218], Programa de Pós-Graduação em Ciências Médicas, Universidade de Brasília, Brasília, Brazil
[219], Anatomía Patológica, Instituto de Investigación Sanitaria San Carlos (IdISSC), Hospital Clínico San Carlos (HCSC), Madrid, Spain
[220], Tecnológico de Monterrey, Monterrey, Mexico
[221], Centro de Investigación en Anomalías Congénitas y Enfermedades Raras (CIACER), Universidad Icesi
[222], Departamento de Genetica, Fundación Valle del Lili
[223], Universidad Complutense de Madrid, Department of Immunology, Ophthalmology and ENT, Madrid, Spain
[224], Department of Neumology, Instituto de Investigación Sanitaria-Fundación Jiménez Díaz University Hospital - Universidad Autónoma de Madrid (IIS-FJD, UAM), Madrid, Spain
[225], Hospital Nuestra Señora de Sonsoles, Ávila, Spain
[226], Inditex, A Coruña, Spain
[227], Intensive Care Department, Instituto de Investigación Sanitaria-Fundación Jiménez Díaz University Hospital - Universidad Autónoma de Madrid (IIS-FJD, UAM), Madrid, Spain
[228], Hospital Universitario Príncipe de Asturias, Servicio de Microbiología Clínica, Madrid, Spain
[229], Universidad de Alcalá de Henares, Departamento de Biomedicina y Biotecnología, Facultad de Medicina y Ciencias de la Salud, Madrid, Spain
[230], GENYCA, Madrid, Spain
[231], Marinha do Brasil, Brazil
[232], Universidade de Brasília, Brasilia, Brazil
[233], Neuromuscular Diseases Unit, Department of Neurology, Hospital de la Santa Creu i Sant Pau, Universitat Autònoma de Barcelona, Barcelona, Spain
[234], Instituto Mexicano del Seguro Social (IMSS), Centro Médico Nacional Siglo XXI, Unidad de Investigación Médica en Enfermedades Infecciosas y Parasitarias, Mexico City, Mexico
[235], Catalan Institution of Research and Advanced Studies (ICREA), Barcelona, Spain
[236], Drug Research Centre, Institut d'Investigació Biomèdica Sant Pau, IIB-Sant Pau, Barcelona, Spain
[237], Departamento de Genetica, Clinica imbanaco
[238], Unidad de Cuidados Intensivos, Hospital Clínico Universitario de Santiago (CHUS), Sistema Galego de Saúde (SERGAS), Santiago de Compostela, Spain
[239], Department of Immunology, Hospital Universitario de Gran Canaria Dr. Negrín, Las Palmas de Gran Canaria, Spain
[240], Department of Clinical Sciences, University Fernando Pessoa Canarias, Las Palmas de Gran Canaria, Spain

[241], University Hospital Germans Trias i Pujol, Pediatrics Department, Badalona, Spain
[242], Department of Pathology, Biobank, Instituto de Investigación Sanitaria-Fundación Jiménez Díaz University Hospital - Universidad Autónoma de Madrid (IIS-FJD, UAM), Madrid, Spain
[243], Faculdade de Ciências da Saúde, Universidade de Brasília, Brasilia, Brazil
[244], Hospital Universitario Virgen de las Nieves, Servicio de Medicina Interna, Granada, Spain
[245], Fundación Universitaria de Ciencias de la Salud, Grupo de Ciencias Básicas en Salud (CBS), Bogotá, Colombia
[246], Sociedad de Cirugía de Bogotá, Hospital de San José, Bogotá, Colombia
[247], Universidad de Granada, Departamento Bioquímica, Biología Molecular e Inmunología III, Granada, Spain
[248], Hospital Infanta Elena, Allergy Unit, Valdemoro, Madrid, Spain
[249], Faculty of Medicine, Universidad Francisco de Vitoria, Madrid, Spain
[250], Hospital Universitario Infanta Leonor, Madrid, Spain
[251], Complutense University of Madrid, Madrid, Spain
[252], Gregorio Marañón Health Research Institute (IiSGM), Madrid, Spain
[253], Reumathology Service, Instituto de Investigación Sanitaria-Fundación Jiménez Díaz University Hospital - Universidad Autónoma de Madrid (IIS-FJD, UAM), Madrid, Spain
[254], Biobank, Puerta de Hierro-Segovia de Arana Health Research Institute, Madrid, Spain
[255], Universidad Rey Juan Carlos, Madrid, Spain
[256], The John Walton Muscular Dystrophy Research Centre, Newcastle University and Newcastle Hospitals NHS Foundation Trust, Newcastle upon Tyne, UK.
[257], Neuromuscular Unit, Neuropediatrics Department, Institut de Recerca Sant Joan de Déu, Hospital Sant Joan de Déu, Spain
[258], Fundació Docència i Recerca Mutua Terrassa, Terrassa, Spain
[259], Casa de Saúde São Lucas, Natal, Brazil
[260], Hospital Rio Grande, Rio Grande do Norte, Natal, Brazil
[261], Intensive Care Unit, Hospital Universitario de Gran Canaria Dr. Negrín, Las Palmas de Gran Canaria, Spain
[262], Universidad Fernando Pessoa Canarias, Las Palmas de Gran Canaria, Spain
[263], Hospital Clinico Universitario de Valladolid, Servicio de Anestesiologia y Reanimación, Valladolid, Spain
[264], Hospital Clinico Universitario de Valladolid, Servicio de Hematologia y Hemoterapia, Valladolid, Spain
[265], Hospital Universitario Lauro Wanderley, Brazil
[266], Hospital Universitario Infanta Leonor, Servicio de Medicina Interna, Madrid, Spain
[267], University Hospital of Burgos, Burgos, Spain
[268], Universidad de Sevilla, Seville, Spain
[269], Fundación Santa Fe de Bogota, Instituto de servicios medicos de Emergencia y trauma, Bogotá, Colombia
[270], Universidad de los Andes, Bogotá, Colombia
[271], Quironprevención, A Coruña, Spain
[272], Junta de Castilla y León, Consejería de Sanidad, Valladolid, Spain
[273], Gerencia Atención Primaria de Burgos, Burgos, Spain
[274], Immunogenetics-Histocompatibility group, Servicio de Inmunología, Instituto de Investigación Sanitaria Puerta de Hierro - Segovia de Arana, Madrid, Spain
[275], Hospital del Mar, Department of Infectious Diseases, Barcelona, Spain
[276], IMIM (Hospital del Mar Medical Research Institute, Institut Hospital del Mar d'Investigacions Mediques), Barcelona, Spain
[277], Universitat Autònoma de Barcelona, Department of Medicine, Spain
[278], Consejería de Sanidad, Comunidad de Madrid, Madrid, Spain
[279], Centro para el Desarrollo de la Investigación Científica, Asunción, Paraguay
[280], Hospital de Infectología, Centro Médico Nacional "La Raza", Instituto Mexicano del Seguro Social (IMSS), Mexico City, Mexico
[281], Genomics Division, Instituto Tecnológico y de Energías Renovables, Santa Cruz de Tenerife, Spain
